# Genome-wide analyses and expression patterns under abiotic stress of *NAC* transcription factors in white pear (*Pyrus bretschneideri*)

**DOI:** 10.1186/s12870-019-1760-8

**Published:** 2019-04-25

**Authors:** Xin Gong, Liangyi Zhao, Xiaofei Song, Zekun Lin, Bingjie Gu, Jinxuan Yan, Shaoling Zhang, Shutian Tao, Xiaosan Huang

**Affiliations:** College of Horticulture, State Key Laboratory of Crop Genetics and Germplasm Enhancement, Nanjing Agricultural University, Nanjing, 210095 China

**Keywords:** Evolutionary pattern, Expression divergence, Gene duplication, *NAC* gene family, Drought stress, Cold stress

## Abstract

**Background:**

Although the genome of Chinese white pear (‘Dangshansuli’) has been released, little is known about the functions, evolutionary history and expression patterns of *NAC* families in this species to date.

**Results:**

In this study, we identified a total of 183 *NAC* transcription factors (*TFs)* in the pear genome, among which 146 pear *NAC* (*PbNAC*) members were mapped onto 16 chromosomes, and 37 *PbNAC* genes were located on scaffold contigs. No *PbNAC* genes were mapped to chromosome 2. Based on gene structure, protein motif analysis, and topology of the phylogenetic tree, the pear *PbNAC* family was classified into 33 groups. By comparing and analyzing the unique *NAC* subgroups in Rosaceae, we identified 19 *NAC* subgroups specific to pear. We also found that whole-genome duplication (WGD)/segmental duplication played critical roles in the expansion of the *NAC* family in pear, such as the 83 *PbNAC* duplicated gene pairs dated back to the two WGD events. Further, we found that purifying selection was the primary force driving the evolution of *PbNAC* family genes. Next, we used transcriptomic data to study responses to drought and cold stresses in pear, and we found that genes in groups C2f, C72b, and C100a were related to drought and cold stress response.

**Conclusions:**

Through the phylogenetic, evolutionary, and expression analyses of the *NAC* gene family in Chinese white pear, we indentified 11 *PbNAC TFs* associated with abiotic stress in pear.

**Electronic supplementary material:**

The online version of this article (10.1186/s12870-019-1760-8) contains supplementary material, which is available to authorized users.

## Background

Transcription factors (*TFs)* are essential for the regulation of gene expression by binding to specific *cis*-acting promoter elements, thereby activating or repressing the rate of transcription of their target gene(s) [1, 2]. *TFs* regulate many biological processes, including cellular morphogenesis, signal transduction, and environmental stress response [3, 4]. The identification and functional characterization of *TFs* are essential for the reconstruction of transcriptional regulatory networks [5]. The name of *NAC* gene family was derived from three transcription factors: (i) *NAM* (no apical meristem), (ii) *ATAF1–2*, and (iii) *CUC2* (cup-shaped cotyledon), all of which had the same DNA-binding domain [6]. *NAC* genes encode plant-specific transcriptional regulators that constitute a large transcription factor family in plants.

Considerable evidence has been collected regarding the functions of *NAC* genes in plant growth and development, and they play a role in enhancing tolerance to abiotic stress, thus aiding adaptation to fluctuating environments [7]. *NAC* genes were first found to be associated with shoot apical meristem and primordium formation in *Petunia* [6]. Later studies identified other *NAC* members, which are involved in transcriptional regulation of diverse biological processes, including shoot apical meristem development [8–10], floral morphogenesis [11], lateral root development [12, 13], leaf senescence [14, 15], stress inducible flowering induction [16, 17], embryogenesis [18], cell cycle control [19–21], cell wall development [22], hormone signaling [23, 24], grain nutrient remobilization [25], and shoot branching determination [22]. In particular, some plant *NAC TFs* have been found to be involved in plant responses to biotic and abiotic stresses [26], including drought, salinity, cold shock, mechanical wounding, and virus infection. The stress-responsive *ANAC096* had a synergistic relationship with ABRE binding factors and increased plant survival rates under osmotic and drought stresses [27, 28]. In addition, *AtNAC019*, *AtNAC055*, and *AtNAC072* specifically bound to *NAC*-recognized sites (*NACRS*) in the promoter of the *EARLY RESPONSIVE TO DEHYDRATION STRESS 1* (*ERD1*) gene to enhance drought tolerance [29]. *Arabidopsis NAC016* repressed transcription of *ABSCISIC ACID-RESPONSIVE ELEMENT BINDING PROTEIN 1* (*AREB1*), a key transcription factor involved in ABA-dependent stress-signaling that facilitates responses to drought stress [27, 30]. In soybean, 38 *NAC* genes were found to be involved in the drought response [31, 32]. *GmNAC11* and *GmNAC20* were linked to different abiotic stresses, and their expression in *Arabidopsis* conferred tolerance to both salt and freezing [33]. Similarly, over-expression of horsegram *MuNAC4* in transgenic groundnut plants reduced damage to membrane structures, and enhanced osmotic adjustment and antioxidative enzyme regulation under drought stress [34]. In wheat, transgenic lines over-expressing *TaNAC69* produced more biomass in the shoots and roots, when grown under stress-inducing conditions [35, 36]. In maize, *ZmNAC41* and *ZmNAC100* were found to be related to the maize defense network [37]. *ZmSNAC1*, a stress-response transcription factor, enhanced tolerance to dehydration in transgenic *Arabidopsis* [38, 39]. Furthermore, the *StNAC* (*Solanum tuberosum*) gene was induced in response to *Phytophthora infestans* infection and the *BnNAC* genes from *Brassica* were induced by drought and cold [40, 41]. However, to date, little is known regarding how plant *NAC TFs* reduced stress tolerance. Moreover, the molecular basis of this function in white pear has not yet been identified.

Pear is one of the most widely distributed fruits in the world, and has great value for commerce and health. However, pear plants frequently experience abiotic stresses, including drought and cold, which limit pear growth and development, and have subsequent effects on pear crop productivity [42]. Therefore, the identification of genetic determinants associated with drought and cold stresses tolerance in pear is important for agricultural development. In plants, several *NAC* *TFs* are transcriptionally induced by drought and cold, but few *NAC TFs* have been functionally characterized in pear. In this study, we identified 183 *PbNAC* genes from the pear genome and carried out phylogenetic analyses to determine the relationships among these genes. Analyses of protein motifs and intron/exon structures provided support for family classification. Further, we identified duplication events that likely contributed to the expansion of the *NAC* family. In addition, RNA-Seq data showed that the expression patterns of *PbNACs* differed in response to drought and cold stress. This study provides an empirical basis for identifying factors that may enhance the tolerance of pear in response to abiotic environmental stress.

## Results

### Identification and classification of *NAC* genes in Rosaceae

**Fig. 1:**
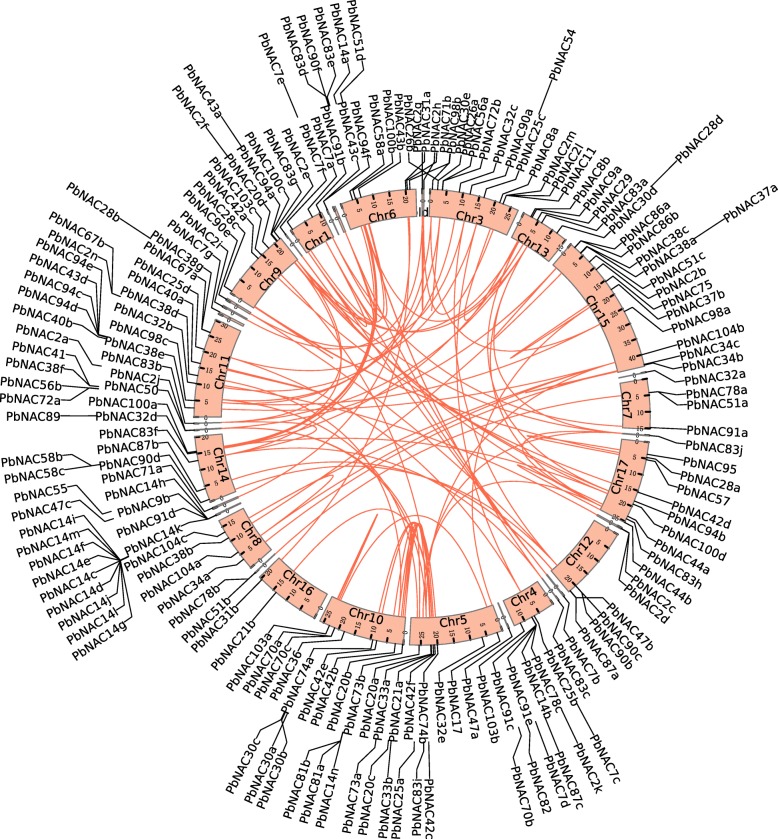
Localization and duplication of the *NAC* genes in the *P. bretschneideri* genome. Circular visualization of *NAC* genes mapped onto different chromosomes using Circos. Chromosome number is indicated on the chromosome. The synteny relationship between each pair of *NAC* genes was detected using linear regression. Genes with a synteny relationship are linked by red lines

To obtain sequences of *NAC* genes for pear and four other Rosaceae species, a HMMER-BLASTP-InterProScan strategy was used to search for genes encoding proteins containing the Pfam PF02365 domain (http://pfam.xfam.org/family/PF02365/). We searched the Chinese white pear genome sequence (http://peargenome.njau.edu.cn/) for genes that encode proteins with *NAC* DNA-binding domains. The protein sequences of *Arabidopsis NAC* were used as queries to perform BLAST searches against the Chinese white pear genome database. In total, we identified 183 *NAC* genes in *Pyrus bretschneideri* (*PbNAC*) (Additional file 1: Table S1). We temporarily named them based on the homologous gene in *Arabidopsis* to better identify individual sequences (Additional file 1: Table S1); for example, *Pbr020642* and *Pbr027956* the homologous gene in *Arabidopsis* is *ANAC002* (e-value of BLAST is 1.45E-151 and 1.62E-122, respectively), and therefore these genes were named *PbNAC2a* and *PbNAC2b*, respectively (Additional file 2: Table S2). Of the 183 *PbNAC* genes, 146 *PbNAC* members were mapped onto 16 chromosomes (excluding chromosome 2); the other 37 *PbNAC* genes were located on scaffold contigs (Fig. 1). Chromosome 11 had the largest number of *PbNAC* genes (20), followed by chromosome 10 with 17 genes. Chromosome 1 was the shortest and contained four genes, while chromosome 7 contained only three. The *PbNAC* genes were clustered in fragments of the chromosome instead of being evenly distributed throughout the chromosome. This may be due to uneven duplication events of pear chromosome fragments [43]. Similarly, we identified 171, 114, 113, and 127 *NAC* genes from the apple (*Malus domestica*), peach (*Prunus persica*), Chinese plum (*Prunus mume*), and strawberry (*Fragaria vesca*) genomes, respectively (Additional file 1: Table S1). As for *PbNAC* genes, their distribution in the four other Rosaceae genomes appeared to be random.

### Gene structure and protein motif analysis of the *NAC* gene family in Chinese white pear

**Fig. 2:**
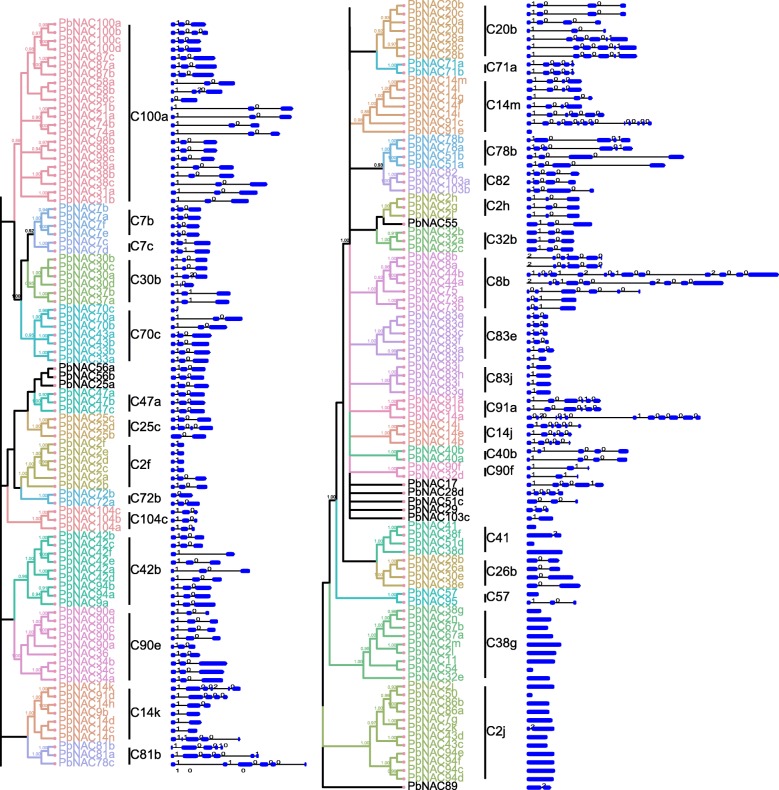
Consensus phylogenetic tree of *NAC* genes in *P. bretschneideri* constructed from amino acid multiple sequence alignment using MrBayes software (left). The numbers beside the branches represent bootstrap values based on 1000 replications. The groups of genes are shown in different colors. Schematic representations of the conserved motifs of *PbNAC* genes (right). Lines connecting two exons represent an intron. NAC domain is marked in bule

To identify the structural diversity of the *PbNAC* genes, we analyzed exon/intron content in the coding sequences of the individual *PbNAC* genes. Of the 183 *PbNAC* genes, 23 had no introns, while most other genes contained at least one (Fig. 2), such as the *PbNAC44b* that had the most (12 introns). A phylogenetic tree was generated from the complete protein sequences of all the *PbNAC* genes, which divided the *NAC* genes into 33 subgroups (Fig. 2). Genes within the same subgroup had a similar exon/intron structure in terms of intron number and exon length. For example, the majority of the *PbNAC* genes in subgroups C100a, C7b, C7c, C30b, C70c, C47a, C104c, C42b, C90e, and C83e had two introns, while all members of subgroups C2f, C83j, and C26b possessed only one intron, with the exception of two members which harbored two. In contrast, members of subgroups C14k, C81b, C20b, C8b, C78b, C91a, C14j, C40b, and C90f had a highly variable member and distribution of introns.

**Fig. 3:**
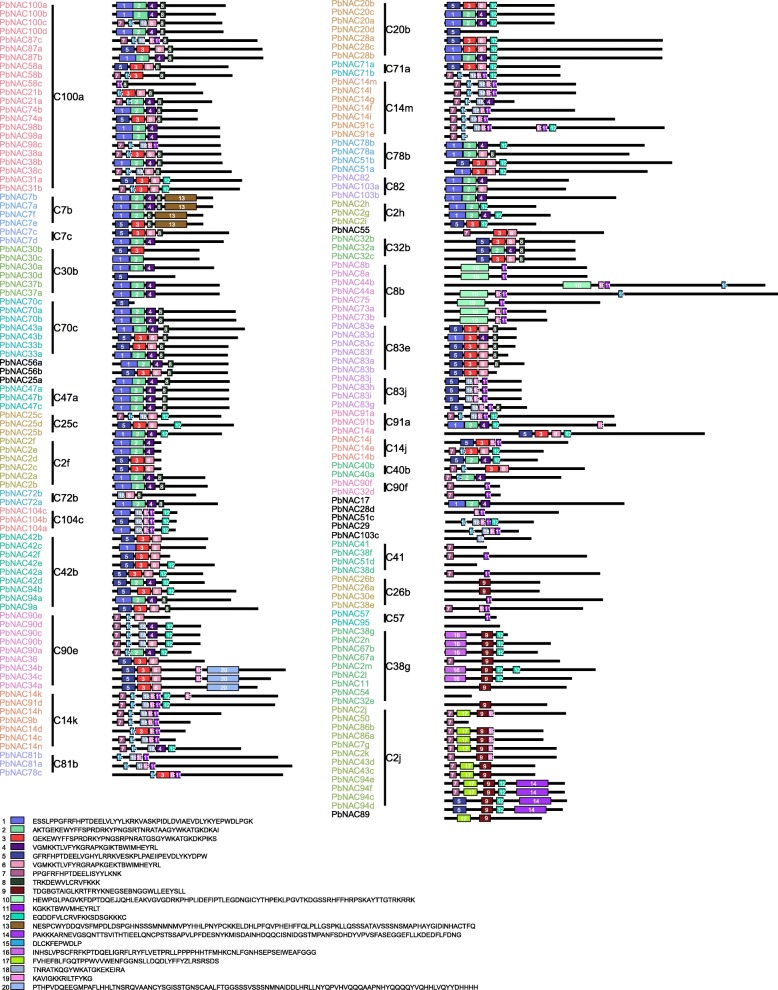
Schematic representations of the conserved motifs and exon-intron compositions. Names of genes are indicated on the left and conserved motifs in NAC proteins on the right. Different motifs are highlighted with different colored boxes with numbers 1 to 20. Lines represent protein regions without detected motif

To further examine the diversity of the *PbNAC* genes, putative motifs were predicted by the program MEME. Based on this program, 20 distinct motifs were identified. This result is similar to that found in apple, which has 19 distinct motifs [44]. As expected, most closely related members had similar motif compositions, which suggested that there are functional similarities among the NAC proteins within the same subgroup (Fig. 3).

### Phylogenetic analysis of pear *NAC* genes

**Fig. 4:**
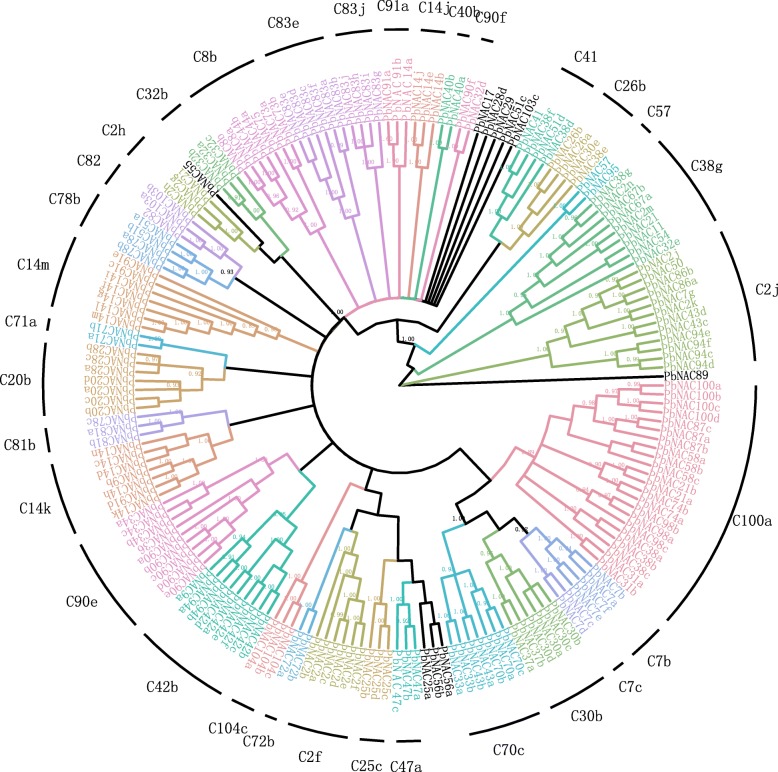
Consensus phylogenetic tree of the *NAC* genes in *P. bretschneideri* constructed from amino acid multiple sequence alignments using MrBayes. The number next to the branch indicates the boot value based on 1000 copies. The different colors mark the groups or subgroups of the *NACs*

**Fig. 5:**
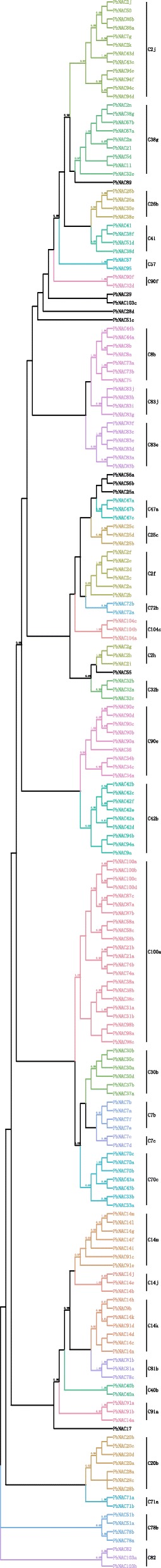
Evolutionary relationship among the pear PbNAC domain sequences. The unrooted tree was generated basing on maximum likelihood analysis. Bootstrap values from 1000 replicates are indicated at each node. The different colors mark the groups or subgroups of the *NACs*

To investigate the phylogenetic relationships between pear *NAC* genes, a rootless phylogenetic tree with 183 complete *NAC* genes from multiple sequence alignments of their NAC domains was constructed. We performed a phylogenetic analysis of Chinese white pear (183 members) *PbNAC* proteins based on *Arabidopsis* (100 members) using the Fast Treeand Mr. Bayes tools. These packages yielded similar results with high support values obtained from both methods (Figs. 4 and 5). All *PbNAC* proteins contained an NAC domain, but their protein structures were highly diverse. The amino acid sequences of NAC proteins were used to construct a phylogenetic tree to investigate the divergence of *PbNAC* proteins. The phylogenetic tree was constructed with conserved N-terminal *NAC* domains A to E, using the same algorithm. The results of this analysis showed that genes in each subgroup may originate from the same duplication event and possess similar functionality. The topology of the phylogenetic tree allowed us to classify the *PbNAC* proteins into 33 subgroups, with most clades having high statistical support (pp > 0.90; bootstrap > 80%; Fig. 4). The internal relationship within the branch was also highly guaranteed (pp = 1.00). We can infer that the genes in each subgroup may originate from the same duplication event, and therefore have similar functions. However, 10 genes, including *PbNAC89*, *PbNAC29*, *PbNAC103c*, *PbNAC28d*, *PbNAC51c*, *PbNAC56a*, *PbNAC56b*, *PbNAC25a*, *PbNAC55*, and *PbNAC17* did not form a cluster in any identified clade or subgroup, therefore the process of genome evolution may have conferred special roles in these *PbNAC* proteins.

**Fig. 6-1 and 6-2:**
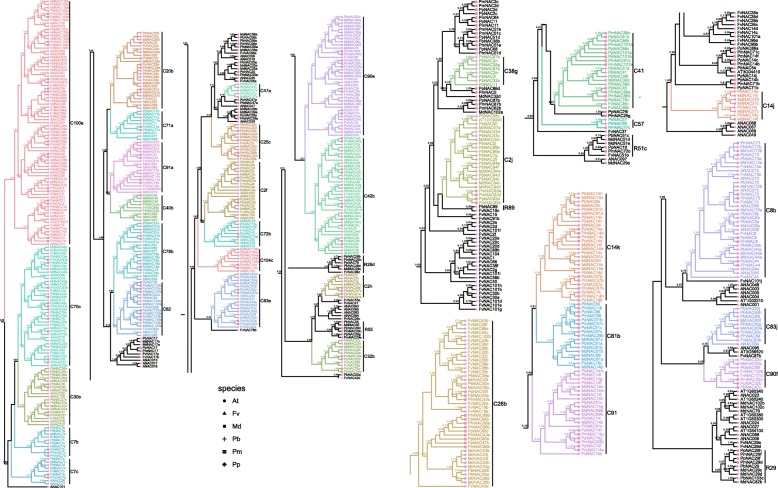
Phylogenetic trees of *NAC* genes in *P. bretschneideri*, *M. domestica*, *P. persica*, *P. mume*, *F. vesca,* and *A. thaliana* based on maximum likelihood analysis of *NAC* domain amino acid sequence alignments. The number next to the branch indicates the boot value based on 1000 copies and different colors mark the subgroups of the *NACs*

### Comparative analysis to reveal unique *NAC* subgroups in Rosaceae

Additional rootless trees were constructed using the same method, which aligned the NAC protein sequences with each of the four Rosaceae species (apple, peach, Chinese plum and strawberry) as well as *Arabidopsis*, as shown in Figs. 6-1 and 6-2. We identified 38 subgroups, including species-specific *NAC* subgroups (R28d, C2h, C25c, C32b, C14k, C26b, C91, R55, C7b, C47a, C14j, C41, R51c, C90f, C81b, R89, R29, C38g, and C57) for which there are no representatives in *Arabidopsis* (Additional file 3: Table S3), suggesting that these proteins might have specialized roles that were either lost in *Arabidopsis*, or were gained after divergence from the last common ancestor. Genes in subgroups C2h, C14k, C81b, and R29 were found in all Rosaceae plants except strawberry (Additional file 3: Table S3). Therefore, we can speculate that these genes may play important roles in woody plants. Furthermore, we discovered that subgroups C47a and C14j were present only in Chinese white pear and apple. We can therefore infer that genes from these subgroups have special functions in Maloideae. Subgroup C38g did not include any *NACs* in *Arabidopsis* or the other four Rosaceae species, but did include *NAC* members from Chinese white pear, indicating that these genes may have been either lost in *Arabidopsis* and the other four Rosaceae species or were acquired in pear after its divergence from the last common ancestor. This evolutionary divergence therefore suggests that these genes may have essential roles in pear.

### Whole-genome duplication and synteny analysis of *PbNAC* genes

It is well documented that gene families have evolved by whole genome duplication, segmental duplication, and tandem duplication, accompanied by post-duplication diversification [45–47]. Genes within a single genome can be classified as singletons, dispersed duplicates, proximal duplicates, tandem duplicates and segmental/WGD duplicates depending on copy number and genomic distribution [48]. In this study, we focused on tandem and segmental/WGD duplications of *NAC TFs* in the whole pear genome. Our results showed that, in Chinese white pear 99 (54.10%) *PbNAC* gene pairs and 116 (63.39%) *PbNAC* genes were duplicated and retained in WGD or segmental events, while only 16 (8.74%) *PbNAC* genes were from gene clusters and 48 (26.23%) *PbNAC* genes originated from tandem duplications (Additional file 4: Table S4). Thus, our results showed the proportion of WGD-type *PbNAC* gene duplication is high in Chinese white pear, which may be due to recent lineage-specific WGD events [30–45 million years ago (MYA)]. With respect to WGD/segmental and tandem duplication events in other Rosaceae plants, Chinese white pear and apple showed different trends from strawberry, plum, and peach. Our results suggest that WGD was the main driver of the expansion of the *NAC* gene family in both apple and Chinese white pear (Additional file 4: Table S4).

In order to investigate the evolutionary mechanisms responsible for the diversity in the *PbNAC* gene family, we identified the mean Ks and duplicated type for each pair of fragments, by using a similar method to that used for the Plant Genome Duplication Database (PGDD). We identified 98 pairs of collinear fragments (Additional file 5: Table S5, Additional file 6: Figures. S1-S2). For example, synteny was detected between fragments *PbNAC73a* and *PbNAC73b*; these showed at least 18 pairs of homologous genes, aligned in the same way. The linear regression coefficient (Q-value) after mapping to a linear regression model was 0.99, showing that the two chromosome segments containing the *NAC* gene were generated by a chromosomal duplication event. The average Ks value of the 18 pairs of homologous genes was 0.04 (standard deviation: 0.03), indicating that this replication event occurred relatively recently. These results further demonstrate that recent lineage-specific WGD events resulted in the expansion of the *PbNAC* gene family in Chinese white pear.

### Historical duplication events

Typically, the evolutionary dates of WGD or segmental duplication events are estimated by Ks values (i.e., synonymous substitutions per site). Previous studies have determined that there are two genome-wide duplication events in the pear genome: an ancient WGD (Ks~ 1.5–1.8) estimated to have taken place approximated 140 MYA [49], and a recent WGD (Ks~ 0.15–0.3) estimated to have taken place at 30–45 MYA [43]. In this study, the evolutionary dates of WGD or segmental duplication events in the *PbNAC* gene family were estimated by Ks values. Additional file 5: Table S5 showed the mean Ks values of *PbNAC* duplicated gene pairs in syntenic regions; Ks values ranged from 0.01 to 7.18. In addition, 88 (89.80%) repeat gene pairs were distributed in two main peaks (Additional file 7: Figure S3). These duplications may derive from the recent WGD (30–45 MYA) and ancient WGD (~ 140 MYA) reported previously, indicating that the *NAC* is an ancient gene family that expand with the recent WGD. In addition, 10 (10.20%) duplicated gene pairs had even higher Ks values (2.88–7.18).

Positive (Darwinian) selection is the process of accumulating new favorable mutations and then spreading throughout the population, while negative (purifying) selection is the process of removing deleterious mutations [50]. In order to detect which selection process drove the evolution of the *PbNAC* gene family in Chinese white pear, coding sequences (CDS) was used to calculate the Ka/Ks ratio of homologs in the *PbNAC* gene family. All Ka/Ks ratios obtained from 84 gene pairs were < 1 (Additional file 8: Table S6), implying that the primary force of the evolution of *PbNAC* family genes was purifying selection. Thus, these 81 genes appear to have undergone a recent WGD/segmental duplication, while eight genes underwent positive selection after replication differentiation (*p* < 0.05) (Additional file 9: Table S7). Moreover, the functionality of these eight genes under positive selection included: nucleic acid binding, DNA binding, molecular function, transcription regulation, biological process, and response to stress.

### Expression of *PbNAC* genes under drought stress

**Fig. 7:**
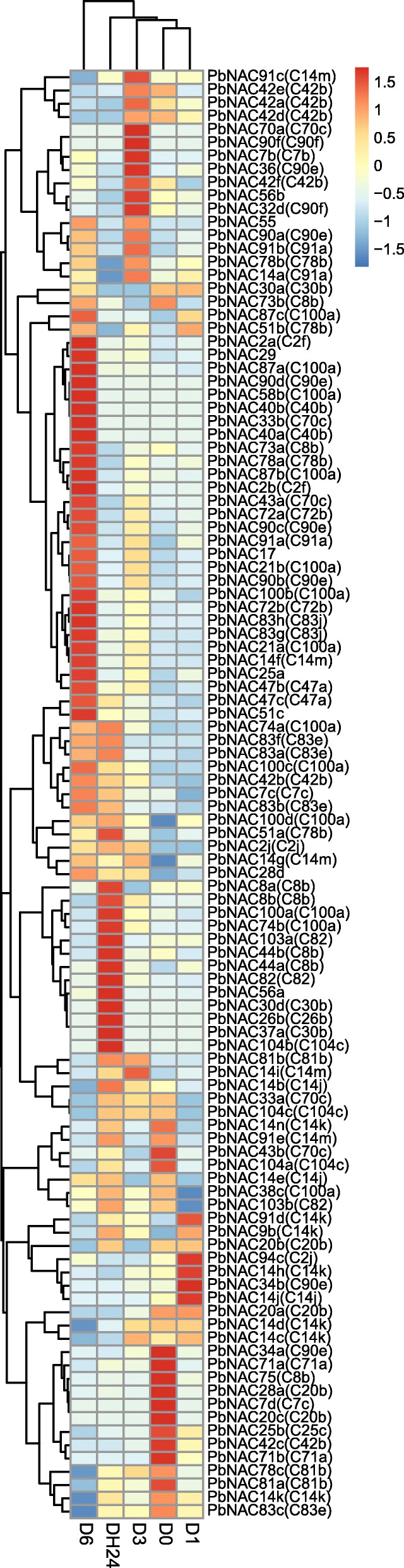
Heat map of RNA-Seq expression of *PbNAC* gene in response to drought. Color scale of the dendrogram represents the scale value of RPKM

Previous studies have reported that numerous NAC domain proteins are implicated in plant drought stress [51–53], however, there is limited information on the response of *NAC TFs* to the drought stress of Chinese white pears. To study the response to drought stress in pear, transcriptomic data of *PbNAC* genes was analyzed in plants exposed to drought treatment. Only 109 *PbNAC* genes showed differential gene expression pattern; six gene clusters were identified and visualized in a heat map (Fig. 7). Cluster 1 contained 16 *PbNAC* genes that experienced significant up-regulation after drought treatment at 3 h. Cluster 2 contained 31 genes that were highly induced at 6 h after drought treatment. Cluster 4 contained 18 genes that were highly induced after re-watering at 24 h, although no transcriptional differences were evident for *PbNAC81b*, *PbNAC14i*, *PbNAC14b*, *PbNAC33a*, or *PbNAC104c*. Cluster 6 contained 23 genes whose transcription significantly increased at 0 and 1 h of drought treatment, while some genes experienced down-regulation at 6 h after drought treatment. Most genes in Clusters 3 and 5 were up-regulated in response to drought treatment, but their relative expression levels were lower than genes in other Clusters. We selected 36 genes that were up-regulated by at least 1.5-fold after drought treatment and studied their expression patterns in other phylogenetic groups (Additional file 10: Table S8).

We found that seven genes in C100a, five genes in C90e, two genes in C47a, C2f, C72b, C8b, C83j, C83e, and C91a, one gene in C42b, C14m, C14j, and C82, and six genes which were not attributed to any group were up-regulated in response to drought stress. Eighteen genes in Cluster 2 were more significantly up-regulated at 6 h than genes in other clusters, and most genes in Cluster 2 belonged to Groups C100a, C2f, C72b, and C90e. These results indicate that water deficiency induced *PbNAC* genes from different groups, and Groups C100a, C2f, C72b, and C90e were primarily involved in biological pathways that mediate drought stress responses.

An orthologous is a homologous gene that differentiates after a speciation event. It is generally assumed that orthologous genes retain the same function in different organisms, and share other key characteristics. Homology pairs (groups) are produced by evolution; ancestral genes and their functions are maintained by speciation events, and mutations may occur within the genes after species differences. In previous studies, 18 *NAC TFs*-including *AtNAC002*, *AtNAC081*, *AtNAC102*, *AtNAC055*, *AtNAC019*, *AtNAC072*, *AtNAC098*, *AtNAC054*, *AtNAC031*, *AtNAC092*, *AtNAC078*, *AtNAC083*, *AtNAC018*, *AtNAC056*, *AtNAC025*, *AtNAC016*, *AtNAC029*, and *AtNAC047* have been shown to be involved in stress responses in *Arabidopsis* [54]. Here we found 46 orthologous *PbNAC TFs*, but only nine *PbNAC* genes, including *PbNAC17*, *PbNAC72a*, *PbNAC72b*, *PbNAC2a*, *PbNAC2b*, *PbNAC100b*, *PbNAC87b*, *PbNAC21a*, and *PbNAC21b* that were up-regulated at 3 or 6 h after drought treatment. These nine stress-response *PbNAC* genes may retain equivalent functions to those found in *Arabidopsis*. In summary, our results indicate that the functions of *PbNAC TFs* are largely conserved.

We next performed a Gene Ontology (GO) analysis of Group C2f, C72b, and C100a, which were the groups with the greatest number of orthologous *PbNAC TFs*. These genes were enriched for the following GO terms: nucleic acid binding, transcription factor activity, multicellular organismal processes, hormone-mediated signaling pathways, developmental processes involved in reproduction, gene expression, post-embryonic root development, transcription regulator activity, response to stress, abscisic acid mediated signaling pathways, response to hormone stimuli, and response to carbohydrate stimuli. Furthermore, Kyoto Encyclopedia of Genes and Genomes (KEGG) pathway enrichment analysis showed that the following terms were associated with these genes: biosynthesis of secondary metabolites, phenylpropanoid biosynthesis, and isoflavonoid biosynthesis. These pathways have previously been shown to be related to abiotic stresses [55, 56].

**Fig. 8:**
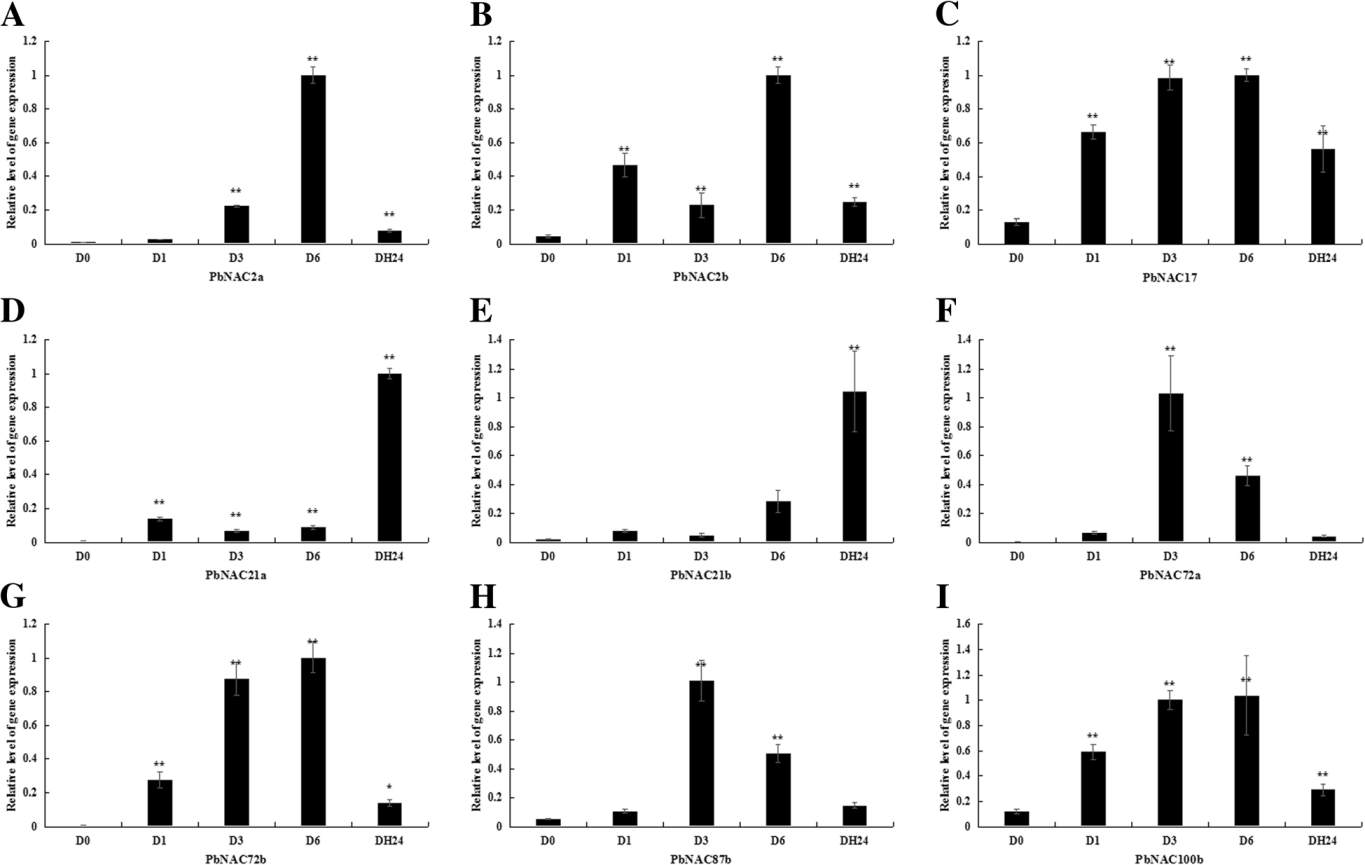
Relative expression of *PbNAC2a* (**a**), *PbNAC2b* (**b**), *PbNAC17* (**c**), *PbNAC21a* (**d**), *PbNAC21b* (**e**), *PbNAC72a* (**f**), *PbNAC72b* (**g**), *PbNAC87b* (**h**) and *PbNAC100b* (**i**) under drought stress. Three biological replicates were used, reference genes were in Additional file 12: Table S10, and bars represent the relative expression of different genes under drought stress. *,**, significant at *P* ≤ 0.05 or 0.01, respectively

To verify that these genes were differentially expressed under drought stress, the relative transcript abundance of nine selected genes were analyzed by quantitative real-time PCR (qRT-PCR). The results of this approach depicted trends in relative gene expression that were largely consistent with the previous approach. Seven genes, including *PbNAC17*, *PbNAC72a*, *PbNAC72b*, *PbNAC2a*, *PbNAC2b*, *PbNAC100b*, and *PbNAC87b*, were up-regulated 3 or 6 h after drought treatment and then down regulated 24 h of recovery. However, our qRT-PCR results for *PbNAC21a* and *PbNAC21b* differed from those shown in the heat map, as they were up-regulated after 24 h of recovery (Fig. 8).

### Expression of *PbNAC* genes under cold treatment

**Fig. 9:**
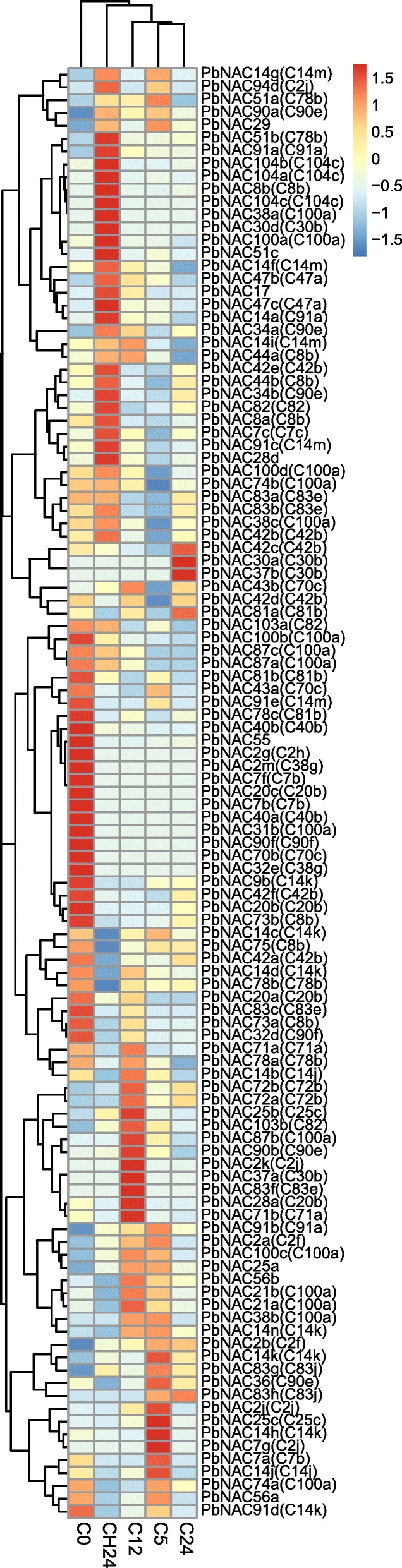
Heat map of RNA-Seq expression of *PbNAC* gene in response to cold. Color scale of the dendrogram represents the scale value of RPKM

We used the same method to analyze the *NAC TFs* in response to cold stress as we did in response to drought stress. Our results suggested that 113 *PbNAC* genes showed differential expression and the resulting heatmap was divided into six clusters (Fig. 9). Cluster 1 (23 genes) and Cluster 2 (20 genes) were significantly up-regulated after returning to room temperature for 24 h. Cluster 3 contained 24 *PbNAC* genes that demonstrated obvious up-regulation at 0 h. In Cluster 5 (20 genes) and Cluster 6 (14 genes), most genes were up-regulated at 5 and 12 h after cold treatment, except *PbNAC74a*, *PbNAC56a*, and *PbNAC91d*. However, after cold treatment some genes including *PbNAC91b*, *PbNAC2b*, *PbNAC83g*, *PbNAC2a*, and *PbNAC100c* were down-regulated at 0 h. The genes in Cluster 4 showed no obvious expression differences in response to cold stress. We selected 26 genes which were up-regulated at least 1.5-fold after cold treatment, to survey the expression patterns in other phylogenetic groups (Additional file 11: Table S9).

**Fig. 10:**
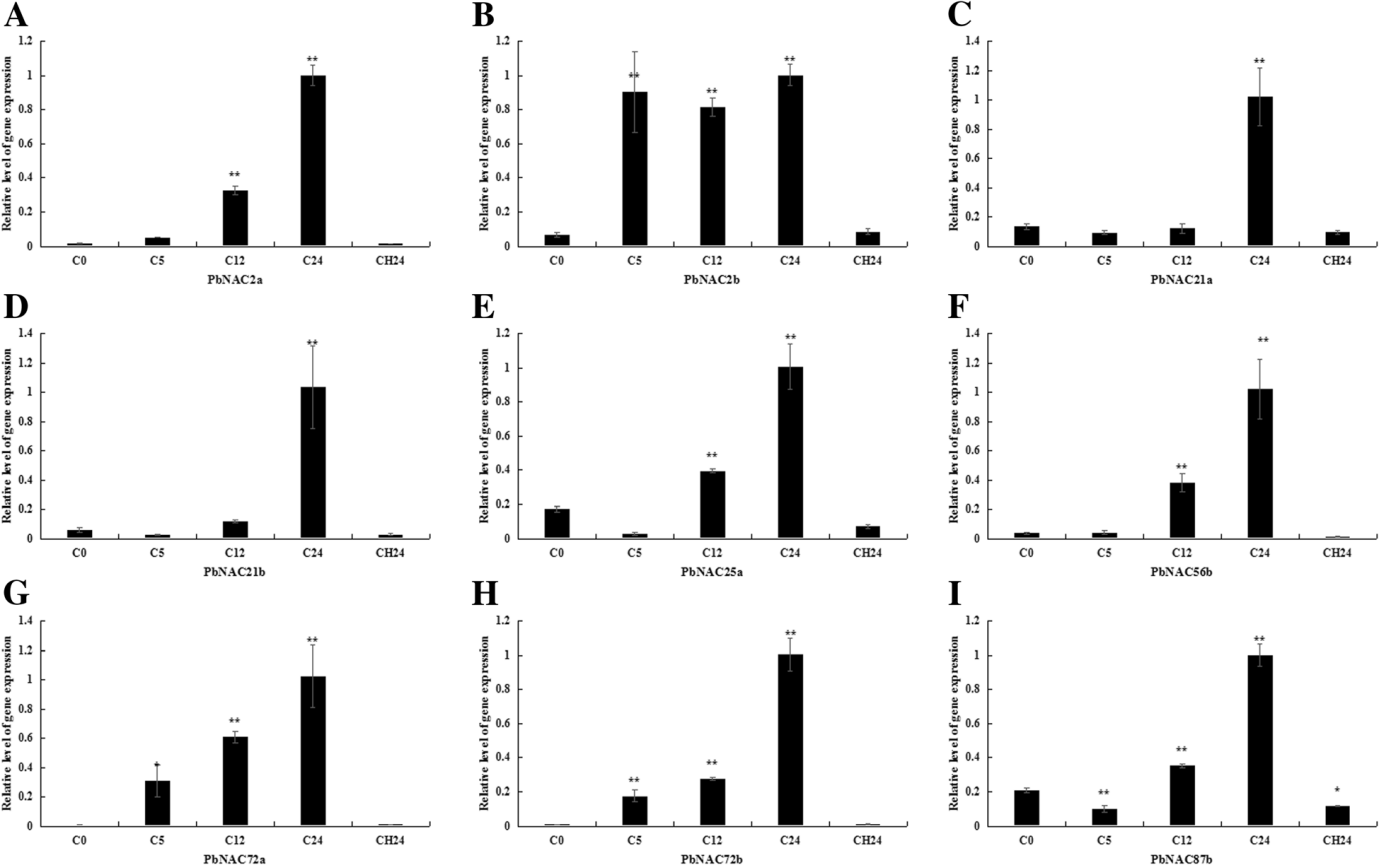
Relative expression of *PbNAC2a* (**a**), *PbNAC2b* (**b**), *PbNAC21a* (**c**), *PbNAC21b* (**d**), *PbNAC25a* (**e**), *PbNAC56b* (**f**), *PbNAC72a* (**g**), *PbNAC72b* (**h**) and *PbNAC87b* (**i**) under cold stress. Three biological replicates were used, reference genes were in Additional file 12: Table S10, and bars represent the relative expression of different genes under cold stress. *,**, significant at P ≤ 0.05 or 0.01, respectively

We found four genes in C100a, three genes in C80b, two genes in C47a, C2f, C72b, C42b, C83e, and C91a, and one gene in C104c, C90e, C83j, and C14m. Moreover, three other genes, which were not assigned to any group, were up-regulated in response to cold stress. Ten genes in Clusters 5 and 6 were more significantly up-regulated at 5 or 12 h, compared to genes in other clusters, and most of these genes belonged to groups C100a, C2f, and C72b. Thus, *PbNAC* genes from separate groups were induced by cold, and these same groups were identified as being involved in biological pathways responding to cold treatment. Similarly, we found seven homologous genes *PbNAC72a*, *PbNAC72b*, *PbNAC2a*, *PbNAC2b*, *PbNAC87b*, *PbNAC21a*, and *PbNAC21b* that were strongly up-regulated not only at 3 or 6 h after drought treatment but also at 5 or 12 h after cold treatment. In addition, *PbNAC56b* and *PbNAC25a* were also found to respond to cold stress. The results of the GO and KEGG pathway analyses were in agreement in this study. To validate the expression patterns of the nine genes in phylogenetic tree groups C100a, C2f, and C72b, we performed qRT-PCR analysis on pear seedlings subjected to short-term cold stress. The results were highly consistent with our RNA-seq data (Fig. 10). The gene expression levels of all nine genes increased to their highest level either at 12 or 24 h, then decreased 24 h after recovery.

## Discussion

As *NAC TFs* play important roles in diverse processes, including developmental programs, defense, and abiotic stress responses, research on these proteins has progressed considerably [57, 58]. Many *NAC TFs* have been identified and functionally characterized in both model and crop plants, including *Arabidopsi*s, rice, soybean, and wheat. Moreover, *NAC TFs* have been identified in apple, but no large dataset of *NAC TFs* exists for Chinese white pear [44].

In this study, we performed genome-wide analyses to identify a total of 183 *PbNAC* genes and larger than 163 in *Populus* [4], 151 in rice [59] and 101 in *Brachypodium distachyon* [60]. The *NAC* gene family is the larger family within plant species, due to an expansion of the *NAC* gene family. It can be speculated that the presence of additional *NAC* genes in the pear genome. Through genome-wide duplication, homologous and historical duplication events, our analyses of the *PbNAC* gene revealed that *NAC* was an ancient gene family that was expanded during the recent WGD. Previous studies have reported a recent WGD incident in China pear and occurred around 30–45 MYA [43, 61]. Su et al. (2013) found the expansion of the apple *NAC* gene occurred in a recent WGD of 60–65 MYA [44] and has been earlier than the pear. In addition, we found evidence that purifiying selection was a major force driving the evolution of the *PbNAC* family of genes.

Based on gene structure and protein motif analyses, the *PbNAC* family was classified into 33 major groups. Motif and exon/intron analyses showed that the most closely related members in the phylogenetic tree had common motif compositions, which points to the existence of functional similarities among *NAC* proteins within the same subfamily. The motif and exon/intron were similar as *Brachypodium distachyon* and apple [44, 60]. To examine the phylogenetic relationships among the *NAC* proteins in Chinese white pear, we identified unique *PbNAC* subgroups in different Rosaceae species and constructed a rootless phylogenetic tree from the alignments of the NAC protein sequences. The different naming and classification methods may be the main reason for some differences between the phylogenetic tree in this study and the phylogenetic tree obtained in previous studies [44, 59, 60]. It could also be explained by different algorithms used in phylogenetic analysis. Although there were some differences, the phylogenetic tree obtained in this study was basically consistent with previous studies. Through the phylogenetic tree, Subgroup C38g was found only in Chinese white pear and members of this group may have specialized roles gained after divergence from closely related species. And the specialized roles of this subgroup need further examine.

Abiotic stresses, such as drought and cold, can cause loss of yield and reduce the quality of fruit trees [62, 63]. The expression of *PbNAC* genes in response to drought and cold stress was investigated by using RNA-Seq data. We found that 36 *PbNAC* genes were up-regulated at least 1.5-fold under drought treatment and 26 *PbNAC* genes under cold treatment. In addition, groups C100a, C2f, and C72b were found to be involved in biological pathways in drought and cold stress responses. Overexpression of *AtNOC019*, *AtNAC055*, *AtNAC016*, *AtNAC096* and *AtNAC072* increase the stress tolerance in *Arabidopsis* [27, 64]. Moreover, *AtNAC019*, *AtNAC055*, and *AtNAC072* could specifically bind to the promoter of *ERD1* of the *NAC* recognition site (NACRS), and enhanced drought tolerance [29]. In rice, 40 *NAC* genes responded to drought or salt stress, and overexpression of *SNAC3* (*ONAC003*), *ONAC022*, *OsNAC5*, *OsNAC2*, and *OsNAC6* improved drought tolerance in transgenic plants [51, 65]. In wheat, overexpression of *TaNAC29*, *TaNAC2*, *TaNAC2a,* and *TaNAC67* was also found to increase tolerance to cold, high salinity, and drought stresses [66, 67]. In this study, 11 *PbNAC TFs* orthologs of *NAC TFs* (identified as being involved in the abiotic stress response) in *Arabidopsis* were found to be responsive to drought and cold stresses in pear. We speculate that these *PbNAC TFs* may have functions in pear that are equivalent to those of their *Arabidopsis* orthologs. Since gene expression patterns can provide important clues for their functions, we examined the expression of selected 11 *PbNAC* genes in drought and cold treatments by using qRT-PCR. Interestingly, these genes are all shown to be involved in the regulation of drought and cold. In future, more researches will be needed to determine the functions of the *PbNAC* genes in pear.

To date, many studies have investigated the important roles of *NAC TFs* in plant growth and development, as well as their response to abiotic stresses. Therefore, the *NAC TF* family has become the subject of continued attention because of its potential involvement in plant tolerance engineering [68–72]. Our results demonstrated that the eleven *PbNAC* genes examined in this study play essential roles in plant responses to abiotic stress, and therefore may be suitable candidate genes for the engineering of pear plants with improved stress resistance. Overall, in this study, we provide a comprehensive analysis of the *NAC* family in Chinese white pear, and we discuss the relationship of this family to abiotic stress responsiveness. The results presented in this report may facilitate the functional characterization of the *NAC* gene family and further understand the relationship among *NAC* family members. Alternatively, our results could also improve our understanding of the molecular basis of important agronomic traits in pear, including fruit development and their abiotic stress response. Taken together, our results may be useful for identifying new candidate *NAC* family genes for genetic engineering of novel pear germplasms with enhanced stress tolerance. We will verify the cold and drought tolerance of these 11 genes in future studies.

## Conclusions

A total of 183 *PbNAC* genes were identified from the pear genome. Based on the phylogenetic relationships between these genes, we divided the 183 *PbNAC* genes into 33 subgroups. The analyses of conserved domains suggested that *PbNAC* genes in the same group had similar functions. *NAC* genes from pear and *Arabidopsis* were present in most subgroups, indicating that these members had a recent common ancestor. Thus, the function of most *NAC* genes might be conserved during angiosperm evolution. Collinearity analysis showed that the recent WGD (30–45 MYA) may have contributed to the large-scale amplification of the *NAC* gene family in Chinese white pear. Purifying selection was found to be major force acting on *NAC* family genes; eight genes underwent positive selection after replication differentiation. Transcriptome sequencing analysis identified *PbNAC* genes likely to play an important role in responses to drought and cold stress. Expression and functional data for *AtNAC* genes supported the hypothesis that *PbNAC* genes possess a variety of unique functions under drought and cold stress. In conclusion, we found eleven *PbNAC* genes that play a key role in the response to abiotic stresses. This will facilitate further research on the biological functions of *NAC TFs* in pear, while the functions of these genes are an important topic for further research on this species.

## Methods

### Identification of *NAC* genes in the white pear genome

The complete genome, proteome sequences, and GFF of pear (*Pyrus bretschneideri*) were obtained from http://peargenome.njau.edu.cn [43]. The corresponding protein sequences in *A. thaliana* were downloaded from the *Arabidopsis* Information Resource (TAIR) (http://www.arabidopsis.org/) [73]. In both proteome datasets, if two or more protein sequences at the same locus were identical, we selected the longest sequence where they overlapped. A HMM profile for the NAM domain (PF02365) was downloaded from Pfam (http://pfam.xfam.org/family/PF02365/). HMMER was used to search a customized database containing the proteome with the threshold set to the Pfam GA gathering cutoff [74]. The HMMER-selected proteins were used for a BLASTp query of the original protein database. Finally, the BLASTp hits were scanned for NAM domains using InterPro (http://www.ebi.ac.uk/interpro/) [75]. We used the same strategies to identify the *NAC* genes from four other Rosaceae plants. The genome sequences of apple, peach, and strawberry were downloaded from Phytozome (http://phytozome.jgi.doe.gov/pz/portal.html#) and the Chinese plum genome sequence was downloaded from the *Prunus mume* Genome Project (http://prunusmumegenome.bjfu.edu.cn) [76].

### Chromosome location, gene structure, and conserved motifs in the *PbNAC* family

We obtained information on the detailed chromosome location of each *PbNAC* gene from genome annotation documents [43]. These data were then integrated and plotted by using Circos software [77]. The gene structure of each *PbNAC* gene was drawn by using the Gene Structure Display Server online software package (http://gsds.cbi.pku.edu.cn/) [78]. To investigate detailed information regarding protein motifs, the MEME tool was used to identify conserved motifs shared among *PbNAC* proteins [79]. The analysis parameters were set as follows: maximum number of different motifs: 30; minimum motif width: 6; and maximum motif width: 50.

### Phylogenetic analysis

We performed multiple sequence alignment on the NAC domains of the protein sequences by using MUSCLE set at default parameters [80]. And we constructed phylogenetic trees by using a Maximum Likelihood approach via FastTree [81]. MrBayes 3.2 was also used to build phylogenetic trees, with parameters set as follows: lset rates = gamma, ngen = 1,000,000, samplefreq = 100, nchains = 4, stopval = 0.01, stoprule = yes and sumtburnin = 100 [82]. Classification of *PbNAC* genes was performed according to their phylogenetic relationship with corresponding *Arabidopsis AtNAC* genes.

### Synteny analysis and tests for positive selection

A method similar to PGDD (http://chibba.agtec.uga.edu/duplication/) was used to perform synteny analysis [83]. First, we identified potential homologous gene pairs where the E-value was less than 1e-5. Next, 15 genes were extracted from the left and right side of *NAC* genes. The minimum number of homologous gene pairs in synteny was 3, and the E-value of the common linear section was 0.01. The Q-value was 0.9. If the alignment of the homologous pairs between these two chromosomal segments were considered to follow a linear relationship (determined by the correlation coefficient of the linear regression), we concluded that there was a collinear relationship between the two fragments, which was presumably due to WGD or segmental duplication. MCScanX downstream analysis tools were used to annotate the Ka and Ks substitution rates of syntenic gene pairs. The average Ks value of homologous pairs in the collinear section reflected the approximate time of replication. We took Ka/Ks < 1 to be indicative of negative selection, while a value equal to 1 indicated neutral selection, and > 1 indicated positive selection. We selected collinear segments where the Ks value was < 0.3 with its sub-family as the background. A program package for phylogenetic analysis by maximum likelihood (PAML) was then used to inspect branch sites [84].

### Illumina sequencing and data analysis

Three-month-old *Pyrus ussuriensis* seedlings were collected from an experimental nursery at National Center of Pear Engineering Technology Research, Nanjing Agricultural University. In order to remove physiological and environment influences, shoots of similar length and age of seedling were chosen. Uniform and healthy plants were carefully and extensively washed and placed in a growth chamber at 26 °C with a 16 h light/8 h dark photoperiod for 2 d. For the drought treatment, the seedlings were put on clean filter papers (90 × 90 mm) and allowed to dry for 0, 1, 3 and 6 h in an ambient environment at 26 °C, followed by recovery in water at 26 °C for 24 h. For the cold treatment, the plants were transferred to 4 °C growth chambers for continuous treatment for 0, 5, 12 and 24 h, followed by recovery at 26 °C for 24 h. For each time point, at least 30 seedlings were used, and the collected samples and stored at − 80 °C until use. Total RNA from samples was isolated as described by Huang et al. (2015) [85]. Solexa/Illumina sequencing was done as described by Qi et al. (2013) [86]. There was one biological replicate in RNA-seq. The raw sequence data analysis and base calling was performed by an Illumina instrument software Analyzer at BGI-Shenzhen. Next, clean sequencing reads were mapped onto the pear genome reference reported by Wu et al. (2013) [43]. An improved ultrafast tool for short read alignment (SOAP) aligner/SOAP2 was subsequently used to identify continuous gene regions [87]. To obtain high quality transcriptomic data, a maximum of two mismatches in clean reads was permitted, and unique mapped reads were used for further analysis. Reads per kb per million reads (RPKM) were used to obtain gene expression levels [88]. Differentially expressed genes (DEGs) were evaluated by using the XYZ R package, with the threshold of significance requiring an FDR ≤0.001 and an absolute value of log_2_^(fold-change)^ ≥ 1. InterPro domains [89] were annotated by InterProScan Release 36.0 [90] to investigate gene expression profiling, and functional assignments were mapped onto GO terms [91]. GO enrichment analysis of genes in this study was performed using WEGO [92]. Enriched KEGG pathways were investigated according to their *P*-values and enrichment factors [93]. This was performed by conducting a BLAST search against the KEGG database, and then indicating the relevant KEGG pathway.

### Real-time PCR

To validate expression patterns obtained by the digital transcript abundance measurements, eleven genes were analyzed using qRT-PCR and the primers were in Additional file 12: Table S10. The RNA samples for digital transcript abundance measurements were also used for qRT-PCR. Total RNA was treated with DNase I to remove genomic DNA contamination. Approximately 1 μg of total RNA was used as a template for reverse transcription using ReverTra Ace- α First Strand cDNA Synthesis Kit (TOYOBO, TOYOBO Biotech, Japan) according to the manufacturers’ instructions. QRT-PCR was performed on a Lightcycler480 (Roche), by using the SYBR® Green Premix kit (TaKaRa Biotechnology, Dalian, China) [43]. The composition of the PCR mix was as follows: 10 μl 2 X SYBR Premix ExTaq™, 2.5 μl each primer, and 1 μl of cDNA template in a final volume of 20 μl [84]. All reactions were run as duplicates in 96-well plates. Three replicates of each technique were used and the data was shown as mean ± standard error (SE) (*n* = 3). Relative expression levels were calculated by using the 2^-ΔΔCt^ method and normalizing to expression of the pear tubulin gene (AB239681) was used as an internal control [94]. The protocol for real-time PCR was as follows: initiation with a 10 min denaturation at 95 °C, followed by 55 cycles of amplification with 15 s of denaturation at 95 °C, 15 s of annealing at 58 °C and 20 s of extension at 72 °C. Reads for fluorescence data collection occurred at 60 °C. A melting curve was performed from 60 to 95 °C to check the specificity of the amplified product [86].

## Additional files

Additional file 1: **Table S1** Basic information of *NAC* genes in five *Rosaceae* species. (XLSX 66 kb)

Additional file 2: **Table S2.** Domains in *NAC* genes in *Pyrus bretschneideri*. (PDF 79 kb)

Additional file 3: **Table S3.** Nineteen unique Rosaceae subgroups. (PDF 57 kb)

Additional file 4: **Table S4.** Numbers of *NAC* genes from WGD/segmental duplication events in five Rosaceae genomes. (PDF 53 kb)

Additional file 5: **Table S5.** Synteny related to genes in the *NAC* gene family in *P. bretschneideri*. (PDF 55 kb)

Additional file 6: **Figure S1.** Coefficients of determination of *PbNAC73a* and *PbNAC73b* .**Figure S2.** Segmental duplication between members of *PbNAC73a* and *PbNAC73b*. (ZIP 25 kb)

Additional file 7: **Figure S3.** Distribution of mean Ks values of *PbNAC* gene pairs in synteny blocks. (PDF 6 kb)

Additional file 8: **Table S6** Ka/Ks related to genes in the *NAC* gene family in *P. bretschneideri*. (PDF 48 kb)

Additional file 9: **Table S7** Positive selection of current WGD/segmental duplication using branch-site model A. (PDF 68 kb)

Additional file 10: **Table S8** Up-regulated genes in response to drought treatment. (XLSX 10 kb)

Additional file 11: **Table S9** Up-regulated genes in response to cold treatment. (XLSX 9 kb)

Additional file 12: **Table S10** Primer details for genes selected for quantitative real-time PCR analysis from results of digital transcript abundance measurements. (PDF 50 kb)

## Abbreviations

AREB1: ABSCISIC ACID-RESPONSIVE ELEMENT BINDING PROTEIN 1
CDS: Coding sequences
DEGs: Differentially expressed genes
ERD1: Early Responsive To Dehydration Stress 1
GO: Gene Ontology
KEGG: Kyoto Encyclopedia of Genes and Genomes
NACRS: NAC-recognized sites
PAML: a program package for phylogenetic analysis by maximum likelihood
PGDD: Plant Genome Duplication Database
qRT-PCR: quantitative real-time PCR
Q-value: the linear regression coefficient
SOAP: an improved ultrafast tool for short read alignment
TFs: Transcription factors
WGD: Whole-genome duplication
