# Supplementary material for: Genome-wide analyses and expression patterns under abiotic stress of NAC transcription factors in white pear (Pyrus bretschneideri)
Source: BMC Plant Biol. 2019 Apr 25;19:161. doi: 10.1186/s12870-019-1760-8 (PMC6485137; doi:10.1186/s12870-019-1760-8)
Supplement: Supplementary file 2 — Table S2. Domains in NAC genes in Pyrus bretschneideri. (PDF 79 kb) [file 12870_2019_1760_MOESM2_ESM.pdf]

Gene	Domain	Domain description	Protein length	Domain start	Domain end	Evalue
PbNAC2a	PF02365	No apical meristem (NAM) protein	296	9	132	3.90E-28
PbNAC2b	PF02365	No apical meristem (NAM) protein	304	13	136	1.20E-27
PbNAC2c	PF02365	No apical meristem (NAM) protein	156	9	132	2.30E-24
PbNAC2d	PF02365	No apical meristem (NAM) protein	156	9	132	2.30E-24
PbNAC2e	PF02365	No apical meristem (NAM) protein	156	9	132	1.10E-23
PbNAC2f	PF02365	No apical meristem (NAM) protein	156	9	132	1.10E-23
PbNAC2g	PF02365	No apical meristem (NAM) protein	338	19	145	1.20E-23
PbNAC2h	PF02365	No apical meristem (NAM) protein	292	19	110	2.50E-14
PbNAC2i	PF02365	No apical meristem (NAM) protein	292	19	110	2.60E-14
PbNAC2j	PF02365	No apical meristem (NAM) protein	387	9	137	1.80E-14
PbNAC2k	PF02365	No apical meristem (NAM) protein	357	11	135	7.90E-12
PbNAC2l	PF02365	No apical meristem (NAM) protein	406	8	151	1.90E-12
PbNAC2m	PF02365	No apical meristem (NAM) protein	481	9	151	2.20E-11
PbNAC2n	PF02365	No apical meristem (NAM) protein	339	10	153	2.50E-11
PbNAC7a	PF02365	No apical meristem (NAM) protein	321	8	135	5.00E-29
PbNAC7b	PF02365	No apical meristem (NAM) protein	321	8	135	5.00E-29
PbNAC7c	PF02365	No apical meristem (NAM) protein	372	9	137	4.00E-28
PbNAC7d	PF02365	No apical meristem (NAM) protein	355	8	135	6.60E-28
PbNAC7e	PF02365	No apical meristem (NAM) protein	290	8	114	1.40E-19
PbNAC7f	PF02365	No apical meristem (NAM) protein	290	8	114	1.40E-19
PbNAC7g	PF02365	No apical meristem (NAM) protein	357	11	136	8.00E-15

PbNAC8a	PF02365	No apical meristem (NAM) protein	455	59	196	2.40E-13
PbNAC8b	PF02365	No apical meristem (NAM) protein	454	59	196	3.30E-14
PbNAC9a	PF02365	No apical meristem (NAM) protein	465	19	148	1.20E-24
PbNAC9b	PF02365	No apical meristem (NAM) protein	249	17	145	1.00E-18
PbNAC11	PF02365	No apical meristem (NAM) protein	389	8	144	1.30E-14
PbNAC14a	PF02365	No apical meristem (NAM) protein	829	236	369	5.70E-27
PbNAC14a	PF12906	RING-variant domain	829	69	110	8.90E-09
PbNAC14b	PF02365	No apical meristem (NAM) protein	297	19	143	7.10E-23
PbNAC14c	PF02365	No apical meristem (NAM) protein	202	21	147	4.70E-20
PbNAC14d	PF02365	No apical meristem (NAM) protein	233	22	149	1.20E-18
PbNAC14e	PF02365	No apical meristem (NAM) protein	316	15	140	7.90E-22
PbNAC14f	PF02365	No apical meristem (NAM) protein	416	17	142	5.60E-24
PbNAC14g	PF02365	No apical meristem (NAM) protein	223	30	155	5.60E-23
PbNAC14h	PF02365	No apical meristem (NAM) protein	347	17	143	6.30E-19
PbNAC14i	PF02365	No apical meristem (NAM) protein	543	19	135	1.10E-22
PbNAC14j	PF02365	No apical meristem (NAM) protein	394	51	178	1.20E-19
PbNAC14k	PF02365	No apical meristem (NAM) protein	528	24	149	1.10E-17
PbNAC14l	PF02365	No apical meristem (NAM) protein	419	31	163	1.70E-20
PbNAC14m	PF02365	No apical meristem (NAM) protein	419	31	163	1.70E-20
PbNAC14n	PF02365	No apical meristem (NAM) protein	410	26	167	8.60E-14
PbNAC17	PF02365	No apical meristem (NAM) protein	573	19	143	1.00E-26
PbNAC20a	PF02365	No apical meristem (NAM) protein	351	7	133	5.60E-29

PbNAC20b	PF02365	No apical meristem (NAM) protein	351	7	133	5.50E-29
PbNAC20c	PF02365	No apical meristem (NAM) protein	351	7	133	5.50E-29
PbNAC20d	PF02365	No apical meristem (NAM) protein	173	7	65	2.10E-07
PbNAC20d	PF01597	Glycine cleavage H-protein	173	50	162	1.40E-31
PbNAC21a	PF02365	No apical meristem (NAM) protein	318	12	134	1.30E-26
PbNAC21b	PF02365	No apical meristem (NAM) protein	289	21	105	4.00E-15
PbNAC25a	PF02365	No apical meristem (NAM) protein	374	16	142	9.30E-28
PbNAC25b	PF02365	No apical meristem (NAM) protein	349	14	140	1.80E-25
PbNAC25c	PF02365	No apical meristem (NAM) protein	347	16	142	2.30E-25
PbNAC25d	PF02365	No apical meristem (NAM) protein	387	16	142	1.80E-24
PbNAC26a	PF02365	No apical meristem (NAM) protein	303	8	144	2.50E-16
PbNAC26b	PF02365	No apical meristem (NAM) protein	305	8	144	5.10E-16
PbNAC28a	PF02365	No apical meristem (NAM) protein	695	7	133	7.60E-30
PbNAC28b	PF02365	No apical meristem (NAM) protein	694	7	133	7.60E-30
PbNAC28c	PF02365	No apical meristem (NAM) protein	694	7	133	7.60E-30
PbNAC28d	PF02365	No apical meristem (NAM) protein	364	15	124	2.00E-08
PbNAC29	PF02365	No apical meristem (NAM) protein	236	43	174	1.40E-14
PbNAC30a	PF02365	No apical meristem (NAM) protein	324	6	133	1.20E-28
PbNAC30b	PF02365	No apical meristem (NAM) protein	278	6	111	5.20E-19
PbNAC30c	PF02365	No apical meristem (NAM) protein	278	6	111	5.20E-19
PbNAC30d	PF02365	No apical meristem (NAM) protein	201	6	50	1.30E-08
PbNAC30e	PF02365	No apical meristem (NAM) protein	505	11	146	2.60E-12

PbNAC31a	PF02365	No apical meristem (NAM) protein	413	26	151	3.40E-27
PbNAC31b	PF02365	No apical meristem (NAM) protein	406	23	148	2.50E-26
PbNAC32a	PF02365	No apical meristem (NAM) protein	417	100	226	6.20E-24
PbNAC32b	PF02365	No apical meristem (NAM) protein	417	100	226	6.20E-24
PbNAC32c	PF02365	No apical meristem (NAM) protein	418	100	224	4.80E-23
PbNAC32d	PF02365	No apical meristem (NAM) protein	179	9	143	1.60E-15
PbNAC32e	PF02365	No apical meristem (NAM) protein	327	10	146	7.90E-13
PbNAC33a	PF02365	No apical meristem (NAM) protein	368	12	140	6.00E-28
PbNAC33b	PF02365	No apical meristem (NAM) protein	370	12	140	4.30E-28
PbNAC34a	PF02365	No apical meristem (NAM) protein	462	31	155	1.40E-24
PbNAC34b	PF02365	No apical meristem (NAM) protein	552	30	154	2.80E-24
PbNAC34c	PF02365	No apical meristem (NAM) protein	505	30	154	2.40E-24
PbNAC36	PF02365	No apical meristem (NAM) protein	294	19	145	4.50E-25
PbNAC37a	PF02365	No apical meristem (NAM) protein	342	9	136	4.20E-29
PbNAC37b	PF02365	No apical meristem (NAM) protein	342	9	136	4.20E-29
PbNAC38a	PF02365	No apical meristem (NAM) protein	347	10	136	1.30E-27
PbNAC38b	PF02365	No apical meristem (NAM) protein	351	10	136	1.50E-27
PbNAC38c	PF01582	TIR domain	380	230	366	1.40E-35
PbNAC38c	PF02365	No apical meristem (NAM) protein	380	10	131	2.80E-20
PbNAC38d	PF02365	No apical meristem (NAM) protein	496	8	141	1.40E-17
PbNAC38e	PF02365	No apical meristem (NAM) protein	441	4	140	6.00E-16
PbNAC38f	PF02365	No apical meristem (NAM) protein	454	8	139	4.20E-17

PbNAC38g	PF02365	No apical meristem (NAM) protein	202	10	152	5.50E-12
PbNAC40a	PF02365	No apical meristem (NAM) protein	372	3	128	1.90E-25
PbNAC40b	PF02365	No apical meristem (NAM) protein	447	19	203	1.30E-23
PbNAC41	PF02365	No apical meristem (NAM) protein	135	8	132	2.40E-14
PbNAC42a	PF02365	No apical meristem (NAM) protein	289	3	129	7.00E-24
PbNAC42b	PF02365	No apical meristem (NAM) protein	305	28	155	3.10E-26
PbNAC42c	PF02365	No apical meristem (NAM) protein	298	28	152	3.50E-25
PbNAC42d	PF02365	No apical meristem (NAM) protein	294	3	137	2.90E-24
PbNAC42e	PF02365	No apical meristem (NAM) protein	326	26	150	1.80E-26
PbNAC42f	PF02365	No apical meristem (NAM) protein	184	26	151	1.20E-26
PbNAC43a	PF02365	No apical meristem (NAM) protein	422	17	144	4.20E-28
PbNAC43b	PF02365	No apical meristem (NAM) protein	400	17	144	1.50E-26
PbNAC43c	PF02365	No apical meristem (NAM) protein	289	17	145	2.10E-13
PbNAC43d	PF02365	No apical meristem (NAM) protein	289	17	145	2.10E-13
PbNAC44a	PF01985	CRS1 / YhbY (CRM) domain	1062	576	645	1.80E-13
PbNAC44a	PF02365	No apical meristem (NAM) protein	1062	56	196	3.60E-12
PbNAC44b	PF07714	Protein tyrosine kinase	1392	2	189	4.10E-38
PbNAC44b	PF02365	No apical meristem (NAM) protein	1392	384	524	5.20E-12
PbNAC44b	PF01985	CRS1 / YhbY (CRM) domain	1392	904	973	4.70E-13
PbNAC47a	PF02365	No apical meristem (NAM) protein	372	9	136	3.70E-27
PbNAC47b	PF02365	No apical meristem (NAM) protein	372	9	136	3.70E-27
PbNAC47c	PF02365	No apical meristem (NAM) protein	373	9	136	3.80E-27

PbNAC50	PF02365	No apical meristem (NAM) protein	77	11	49	9.60E-07
PbNAC51a	PF02365	No apical meristem (NAM) protein	647	37	162	2.30E-26
PbNAC51b	PF02365	No apical meristem (NAM) protein	725	38	163	2.70E-26
PbNAC51c	PF02365	No apical meristem (NAM) protein	281	14	141	5.30E-14
PbNAC51d	PF02365	No apical meristem (NAM) protein	104	9	90	2.00E-09
PbNAC54	PF02365	No apical meristem (NAM) protein	87	8	77	2.60E-04
PbNAC55	PF02365	No apical meristem (NAM) protein	508	61	230	2.40E-21
PbNAC56a	PF02365	No apical meristem (NAM) protein	370	32	158	3.40E-28
PbNAC56b	PF02365	No apical meristem (NAM) protein	368	29	155	5.60E-28
PbNAC57	PF02365	No apical meristem (NAM) protein	166	10	143	7.00E-14
PbNAC58a	PF02365	No apical meristem (NAM) protein	370	6	130	1.50E-28
PbNAC58b	PF02365	No apical meristem (NAM) protein	383	6	99	2.20E-16
PbNAC58c	PF02365	No apical meristem (NAM) protein	266	1	27	8.60E-05
PbNAC67a	PF02365	No apical meristem (NAM) protein	368	9	153	1.80E-12
PbNAC67b	PF02365	No apical meristem (NAM) protein	297	10	153	1.20E-11
PbNAC70a	PF02365	No apical meristem (NAM) protein	393	10	137	7.60E-28
PbNAC70b	PF02365	No apical meristem (NAM) protein	395	10	137	5.00E-28
PbNAC70c	PF02365	No apical meristem (NAM) protein	71	10	52	5.20E-09
PbNAC71a	PF02365	No apical meristem (NAM) protein	369	7	133	5.20E-29
PbNAC71b	PF02365	No apical meristem (NAM) protein	369	7	133	5.10E-29
PbNAC72a	PF02365	No apical meristem (NAM) protein	336	15	139	1.90E-25
PbNAC72b	PF02365	No apical meristem (NAM) protein	267	16	70	1.60E-08

PbNAC73a	PF02365	No apical meristem (NAM) protein	324	57	196	1.70E-15
PbNAC73b	PF02365	No apical meristem (NAM) protein	327	56	195	1.70E-15
PbNAC74a	PF02365	No apical meristem (NAM) protein	272	11	137	7.60E-28
PbNAC74b	PF02365	No apical meristem (NAM) protein	272	11	137	1.50E-27
PbNAC75	PF02365	No apical meristem (NAM) protein	496	47	188	2.60E-16
PbNAC78a	PF02365	No apical meristem (NAM) protein	589	11	136	6.70E-28
PbNAC78b	PF02365	No apical meristem (NAM) protein	637	11	136	7.40E-28
PbNAC78c	PF02365	No apical meristem (NAM) protein	544	88	212	6.70E-15
PbNAC81a	PF02365	No apical meristem (NAM) protein	573	4	132	5.50E-11
PbNAC81b	PF02365	No apical meristem (NAM) protein	528	31	111	2.20E-06
PbNAC82	PF02365	No apical meristem (NAM) protein	396	9	135	5.80E-25
PbNAC83a	PF02365	No apical meristem (NAM) protein	255	15	138	1.90E-26
PbNAC83b	PF02365	No apical meristem (NAM) protein	167	15	138	3.80E-27
PbNAC83c	PF02365	No apical meristem (NAM) protein	226	15	138	4.40E-25
PbNAC83d	PF02365	No apical meristem (NAM) protein	230	15	138	4.60E-25
PbNAC83e	PF02365	No apical meristem (NAM) protein	230	15	138	4.60E-25
PbNAC83f	PF02365	No apical meristem (NAM) protein	203	15	138	2.20E-25
PbNAC83g	PF02365	No apical meristem (NAM) protein	263	17	152	5.10E-23
PbNAC83h	PF02365	No apical meristem (NAM) protein	246	2	134	5.00E-21
PbNAC83i	PF02365	No apical meristem (NAM) protein	246	2	134	5.00E-21
PbNAC83j	PF02365	No apical meristem (NAM) protein	246	2	134	5.00E-21
PbNAC86a	PF02365	No apical meristem (NAM) protein	315	9	137	1.10E-13

PbNAC86b	PF02365	No apical meristem (NAM) protein	315	9	137	1.10E-13
PbNAC87a	PF02365	No apical meristem (NAM) protein	478	27	165	5.70E-29
PbNAC87b	PF02365	No apical meristem (NAM) protein	481	27	165	5.40E-29
PbNAC87c	PF02365	No apical meristem (NAM) protein	462	27	149	6.60E-24
PbNAC89	PF02365	No apical meristem (NAM) protein	310	8	144	7.40E-12
PbNAC90a	PF02365	No apical meristem (NAM) protein	252	5	134	2.50E-23
PbNAC90b	PF02365	No apical meristem (NAM) protein	281	5	141	1.80E-22
PbNAC90c	PF02365	No apical meristem (NAM) protein	280	5	141	8.30E-22
PbNAC90d	PF02365	No apical meristem (NAM) protein	283	5	141	2.10E-21
PbNAC90e	PF02365	No apical meristem (NAM) protein	185	5	99	2.10E-13
PbNAC90f	PF02365	No apical meristem (NAM) protein	177	9	141	1.60E-17
PbNAC91a	PF02365	No apical meristem (NAM) protein	541	10	135	1.30E-26
PbNAC91b	PF02365	No apical meristem (NAM) protein	546	15	140	4.30E-27
PbNAC91c	PF02365	No apical meristem (NAM) protein	701	19	146	2.20E-19
PbNAC91d	PF02365	No apical meristem (NAM) protein	518	23	148	1.30E-14
PbNAC91e	PF02365	No apical meristem (NAM) protein	74	19	62	2.10E-07
PbNAC94a	PF02365	No apical meristem (NAM) protein	378	16	141	1.20E-25
PbNAC94b	PF02365	No apical meristem (NAM) protein	395	16	156	8.90E-25
PbNAC94c	PF02365	No apical meristem (NAM) protein	390	26	152	7.60E-16
PbNAC94d	PF02365	No apical meristem (NAM) protein	377	26	152	5.50E-16
PbNAC94e	PF02365	No apical meristem (NAM) protein	383	26	158	4.70E-15
PbNAC94f	PF02365	No apical meristem (NAM) protein	383	26	158	9.10E-15

PbNAC95	PF02365	No apical meristem (NAM) protein	177	10	122	6.90E-08
PbNAC98a	PF02365	No apical meristem (NAM) protein	343	16	142	1.10E-28
PbNAC98b	PF02365	No apical meristem (NAM) protein	343	16	142	1.10E-28
PbNAC98c	PF02365	No apical meristem (NAM) protein	345	17	143	8.00E-29
PbNAC100a	PF02365	No apical meristem (NAM) protein	361	18	142	3.60E-29
PbNAC100b	PF02365	No apical meristem (NAM) protein	330	18	142	1.40E-28
PbNAC100c	PF02365	No apical meristem (NAM) protein	350	17	141	3.20E-30
PbNAC100d	PF02365	No apical meristem (NAM) protein	354	17	141	1.10E-29
PbNAC103a	PF02365	No apical meristem (NAM) protein	387	10	135	2.60E-24
PbNAC103b	PF02365	No apical meristem (NAM) protein	547	8	133	8.60E-23
PbNAC103c	PF02365	No apical meristem (NAM) protein	277	29	170	1.90E-06
PbNAC104a	PF02365	No apical meristem (NAM) protein	202	9	131	3.00E-24
PbNAC104b	PF02365	No apical meristem (NAM) protein	205	9	131	9.20E-23
PbNAC104c	PF02365	No apical meristem (NAM) protein	207	9	131	9.50E-23
