# Supplementary material for: Genome-wide analyses and expression patterns under abiotic stress of NAC transcription factors in white pear (Pyrus bretschneideri)
Source: BMC Plant Biol. 2019 Apr 25;19:161. doi: 10.1186/s12870-019-1760-8 (PMC6485137; doi:10.1186/s12870-019-1760-8)
Supplement: Supplementary file 3 — Table S3. Nineteen unique Rosaceae subgroups. (PDF 57 kb) [file 12870_2019_1760_MOESM3_ESM.pdf]

| Unique subgroups in Rosaceas | | | | | | |
|---|---|---|---|---|---|---|
| **Subgro** | **P. bretschneideri** | **A. thaliana** | **M. domestica** | **P. persica** | **P. mume** | **F. vesca** |
| R28d | PbNAC28d | Na | MdNAC28c | PpNAC28b, PpNAC79 | PmNAC28c | FvNAC96c |
| C2h | PbNAC2i, PbNAC2h, PbNAC2g | Na | MdNAC47c | PpNAC29e, PpNAC81a | PmNAC47 b, PmNAC81a | Na |
| C25c | PbNAC25c, PbNAC25d, PbNAC25b | Na | MdNAC25d, MdNAC25e, MdNAC25c | PpNAC29b, PpNAC56b | PmNAC56 b, PmNAC25 | FvNAC2c, FvNAC25b |
| C32b | PbNAC32c, PbNAC32b, PbNAC32a | Na | MdNAC32b, MdNAC32a | PpNAC32a, PpNAC29c | PmNAC32a , PmNAC29 | FvNAC29b, FvNAC29a |
| C14k | PbNAC14h, PbNAC9b, PbNAC91d, PbNAC14k, PbNAC14n, PbNAC14c, PbNAC14d | Na | MdNAC14o, MdNAC9c, MdNAC91d, MdNAC54, MdNAC14i, MdNAC14d, MdNAC91c, MdNAC50, MdNAC14c, MdNAC14e | PpNAC14e, PpNAC14d, PpNAC57b, PpNAC57d, PpNAC14i, PpNAC78b | PmNAC14 d, PmNAC14c , PmNAC57 b, PmNAC57a , PmNAC14 h | Na |
| C26b | PbNAC38e, PbNAC30e, PbNAC26b, PbNAC26a | Na | MdNAC43l, MdNAC30c, MdNAC43k, MdNAC43j, MdNAC43i, MdNAC43h, MdNAC43g, MdNAC26a, MdNAC26b | PpNAC43f, PpNAC43e, PpNAC66b, PpNAC66c, PpNAC66a, PpNAC47b | PmNAC43 d, PmNAC66 d, PmNAC66a , PmNAC66c , PmNAC43e , PmNAC66 b | FvNAC43d, FvNAC28f, FvNAC86a, FvNAC30c, FvNAC102b, FvNAC20b, FvNAC38d, FvNAC86b, FvNAC32b, FvNAC32c, FvNAC55b, FvNAC38e, FvNAC38b, FvNAC38c, FvNAC32a, FvNAC19b, FvNAC19a, FvNAC43e |

| | | | | | | |
|---|---|---|---|---|---|---|
| C91 | PbNAC14m, PbNAC14l, PbNAC14g, PbNAC14f, PbNAC91c, PbNAC14i, PbNAC91e | Na | MdNAC14n, MdNAC14m, MdNAC14l, MdNAC14h, MdNAC14g, MdNAC69a, MdNAC69b, MdNAC14k | PpNAC14h, PpNAC14g, PpNAC14f | PmNAC14g, PmNAC62a, PmNAC14e | FvNAC14b |
| R55 | PbNAC55 | Na | MdNAC55 | PpNAC29d | PmNAC29c | Na |
| C7b | PbNAC7b, PbNAC7a, PbNAC7f, PbNAC7e | Na | MdNAC7b, MdNAC7d | PpNAC7b | PmNAC7c, PmNAC7b | FvNAC7a |
| C47a | PbNAC47a, PbNAC47b, PbNAC47c | Na | MdNAC47a, MdNAC47b | Na | Na | Na |
| C14j | PbNAC14e, PbNAC14j, PbNAC14b | Na | MdNAC14j, MdNAC57d, MdNAC57c, MdNAC51c, MdNAC14f | Na | Na | Na |
| C41 | PbNAC51d, PbNAC41, PbNAC38f, PbNAC38d | Na | MdNAC38c | PpNAC81b, PpNAC86b, PpNAC86c, PpNAC101a, PpNAC101b, PpNAC45, PpNAC78c | PmNAC86c, PmNAC101b, PmNAC81b, PmNAC101a, PmNAC86a | FvNAC36c, FvNAC36b |
| R51c | PbNAC51c | Na | MdNAC51d, MdNAC51e | PpNAC19 | PmNAC72b | FvNAC51b |
| C90f | PbNAC32d, PbNAC90f | Na | MdNAC32c | PpNAC32b, PpNAC47c | PmNAC32b | FvNAC102a, FvNAC101c |
| C81b | PbNAC81a, PbNAC81b, PbNAC78c | Na | MdNAC81c, MdNAC81b, MdNAC86, MdNAC81a, MdNAC14p | PpNAC50, PpNAC96, PpNAC51b, PpNAC51c, PpNAC57c | PmNAC14i, PmNAC96, PmNAC57c, [illegible] | Na |
| R89 | PbNAC89 | Na | Na | Na | Na | FvNAC19c |
| R29 | PbNAC29, PbNAC103c | Na | MdNAC29c, MdNAC29d, MdNAC82b | PpNAC29h, PpNAC29f | PmNAC29f, PmNAC29 | Na |

| | | | | | | |
|---|---|---|---|---|---|---|
| C38g | PbNAC67b, PbNAC2n, PbNAC38g, PbNAC67a, PbNAC2m, PbNAC2l, PbNAC32e, PbNAC11 | Na | Na | Na | Na | Na |
| C57 | PbNAC57, PbNAC95 | Na | Na | Na | PmNAC47c | Na |
