# Supplementary material for: Genome-wide analyses and expression patterns under abiotic stress of NAC transcription factors in white pear (Pyrus bretschneideri)
Source: BMC Plant Biol. 2019 Apr 25;19:161. doi: 10.1186/s12870-019-1760-8 (PMC6485137; doi:10.1186/s12870-019-1760-8)
Supplement: Supplementary file 4 — Table S4. Numbers of NAC genes from WGD/segmental duplication events in five Rosaceae genomes. (PDF 53 kb) [file 12870_2019_1760_MOESM4_ESM.pdf]

Numbers of NAC genes from WGD/segmental duplication events in five Rosaceae genomes					
		**WGD/segmental duplication**		**Tandem duplication**	
**Species**	**Number of NAC genes**	**No. of gene pairs**	**No. of genes**	**No. of cluster**	**No. of genes**
Pyrus	183	99 (54.10%)	116 (63.39%)	16 (8.74%)	48 (26.23%)
Malus domestica	171	91 (53.22%)	104 (60.82%)	16 (9.36%)	49 (28.65%)
Prunus persica	114	18 (15.79%)	30 (26.32%)	12 (10.53%)	36 (31.58%)
Prunus mume	113	11 (9.73%)	17 (15.04%)	14 (12.39%)	41 (36.28%)
Fragaria vesca	127	7 (5.51%)	14 (11.02%)	13 (10.24%)	32 (25.20%)
