# Supplementary material for: Genome-wide analyses and expression patterns under abiotic stress of NAC transcription factors in white pear (Pyrus bretschneideri)
Source: BMC Plant Biol. 2019 Apr 25;19:161. doi: 10.1186/s12870-019-1760-8 (PMC6485137; doi:10.1186/s12870-019-1760-8)
Supplement: Supplementary file 5 — Table S5. Synteny related to genes in the NAC gene family in P. bretschneideri. (PDF 55 kb) [file 12870_2019_1760_MOESM5_ESM.pdf]

Synteny related to genes in NAC gene family in P. bretschneideri					
**Duplicated gene 1**	**Duplicated gene 2**	**Number of homologs**	**E value**	**Mean of Ks**	**standard deviation of Ks**
PbNAC83j	PbNAC83h	23	5.19E-33	0.01	0.01
PbNAC58a	PbNAC58c	24	6.94E-33	0.03	0.02
PbNAC42a	PbNAC42d	24	1.88E-29	0.05	0.04
PbNAC7f	PbNAC7e	22	4.07E-29	0.02	0.03
PbNAC8a	PbNAC8b	20	5.66E-27	0.04	0.03
PbNAC73a	PbNAC73b	18	2.14E-24	0.04	0.03
PbNAC2j	PbNAC86b	18	3.65E-23	0.2	0.81
PbNAC7a	PbNAC7e	17	4.61E-22	0.2	0.54
PbNAC37b	PbNAC37a	14	5.01E-22	0.01	0.01
PbNAC44b	PbNAC44a	16	8.35E-22	0.01	0.01
PbNAC7f	PbNAC7a	17	9.8E-22	0.22	0.66
PbNAC83g	PbNAC83h	14	2.35E-21	0.04	0.03
PbNAC30a	PbNAC30b	14	7.05E-21	0.52	1.87
PbNAC7b	PbNAC7e	16	1.2E-20	0.11	0.2
PbNAC20b	PbNAC20c	15	1.69E-20	0.34	0.76
PbNAC7a	PbNAC7b	16	1.72E-20	0.01	0.03
PbNAC71b	PbNAC71a	16	4.61E-20	0.01	0.01
PbNAC95	PbNAC57	12	4.89E-20	0.01	0
PbNAC83g	PbNAC83j	14	6E-20	0.03	0.02
PbNAC7f	PbNAC7b	15	9.83E-20	0.09	0.23
PbNAC74a	PbNAC74b	14	1.04E-19	0.04	0.02
PbNAC100c	PbNAC100d	13	4.79E-19	0.06	0.08
PbNAC94a	PbNAC94b	13	6.42E-19	0.05	0.04
PbNAC33b	PbNAC33a	16	1.44E-18	0.4	1.35
PbNAC32d	PbNAC90f	13	1.85E-18	0.04	0.03
PbNAC90e	PbNAC90c	12	2.59E-18	0.04	0.03
PbNAC34c	PbNAC34b	12	2.82E-18	2.69	3.68
PbNAC98b	PbNAC98a	11	4.28E-18	0.02	0.03
PbNAC20a	PbNAC20b	13	7.51E-18	0.04	0.04
PbNAC20a	PbNAC20c	11	1.18E-17	0.06	0.06
PbNAC98b	PbNAC98c	13	3.96E-17	0.12	0.17
PbNAC2j	PbNAC86a	13	4.08E-17	0.03	0.04
PbNAC98c	PbNAC98a	12	4.09E-17	0.06	0.07
PbNAC91b	PbNAC91a	15	2.14E-16	0.06	0.05
PbNAC103a	PbNAC82	13	5.09E-16	0.04	0.02
PbNAC104a	PbNAC104c	11	1.36E-15	0.03	0.02
PbNAC31b	PbNAC31a	12	9.8E-15	0.05	0.04
PbNAC28a	PbNAC28c	10	8.15E-14	0.03	0.01
PbNAC87b	PbNAC87a	9	3.67E-13	0.11	0.18
PbNAC87b	PbNAC47b	9	4.72E-13	0.11	0.18
PbNAC42f	PbNAC42e	10	5.04E-13	0.37	1.05
PbNAC25d	PbNAC25c	11	1.28E-12	0.06	0.11
PbNAC38b	PbNAC38a	8	2.91E-12	0.08	0.06

PbNAC83a	PbNAC83b	8	1.22E-11	0.05	0.05
PbNAC40a	PbNAC40b	8	3.81E-11	1.06	2.95
PbNAC100b	PbNAC100a	8	6.01E-11	0.03	0.01
PbNAC32b	PbNAC32c	7	2.25E-10	0.06	0.07
PbNAC90d	PbNAC90c	7	5.77E-10	0.18	0.24
PbNAC28a	PbNAC28b	6	1.8E-09	0.03	0.01
PbNAC83i	PbNAC83h	5	2.53E-09	0.01	0
PbNAC104b	PbNAC104c	5	4.68E-09	0.01	0
PbNAC83j	PbNAC83i	5	7.03E-09	0.02	0
PbNAC86a	PbNAC86b	5	4.16E-08	0.39	0.62
PbNAC104a	PbNAC104b	6	5.06E-08	0.04	0.02
PbNAC83c	PbNAC83d	5	0.000000203	0.01	0
PbNAC2g	PbNAC2h	6	0.000000207	1.87	3.2
PbNAC94b	PbNAC9a	7	0.000000336	1.36	1.72
PbNAC83c	PbNAC83e	5	0.000000561	0.01	0
PbNAC25c	PbNAC25b	5	0.00000414	1.97	2.74
PbNAC56a	PbNAC72a	4	0.00000478	0.04	0.02
PbNAC56a	PbNAC56b	4	0.00000602	0.04	0.02
PbNAC29	PbNAC32d	5	0.00000723	0.39	0.25
PbNAC25d	PbNAC25b	4	0.00000982	2.04	1.4
PbNAC38d	PbNAC42b	3	0.0000158	0.03	0.01
PbNAC51b	PbNAC78a	5	0.0000189	0.03	0.03
PbNAC90a	PbNAC90c	3	0.0000207	0.66	0.71
PbNAC83f	PbNAC83d	3	0.0000238	0.09	0.07
PbNAC2h	PbNAC2m	4	0.0000319	4.04	4.09
PbNAC83f	PbNAC83e	3	0.0000397	0.13	0.14
PbNAC30a	PbNAC30c	4	0.0000424	1.77	3.5
PbNAC42c	PbNAC50	4	0.0000485	5.79	5.99
PbNAC83g	PbNAC83a	4	0.0000796	3.5	3.85
PbNAC90a	PbNAC90e	3	0.0000929	0.29	0.11
PbNAC29	PbNAC90f	4	0.000105	0.5	0.44
PbNAC83c	PbNAC83f	4	0.000134	0.1	0.13
PbNAC56a	PbNAC72b	4	0.00016	0.14	0.12
PbNAC21a	PbNAC21b	4	0.000179	2.3	3.91
PbNAC25a	PbNAC56b	3	0.000186	0.32	0.08
PbNAC25a	PbNAC72a	3	0.000212	0.32	0.08
PbNAC42c	PbNAC42b	3	0.000359	0.08	0.03
PbNAC21a	PbNAC2j	4	0.000504	7.18	5.44
PbNAC83a	PbNAC83j	4	0.000518	2.31	4.01
PbNAC91b	PbNAC14a	5	0.000584	1.78	1.4
PbNAC2a	PbNAC2c	3	0.000865	0.21	0.31
PbNAC104b	PbNAC38e	3	0.000867	6.72	1.78
PbNAC2g	PbNAC2i	3	0.00108	0.01	0.01
PbNAC34a	PbNAC34c	3	0.00127	0.04	0.02
PbNAC100c	PbNAC100a	4	0.00127	2.08	2.61
PbNAC43b	PbNAC43a	3	0.00135	2.67	4.08
PbNAC42c	PbNAC104b	3	0.00139	3.92	5.05
PbNAC83f	PbNAC83b	3	0.00145	4.86	6.42

PbNAC83a	PbNAC83h	3	0.00148	2.88	4.69
PbNAC21a	PbNAC103c	4	0.00157	5.11	3.16
PbNAC7d	PbNAC37a	3	0.00253	0.46	0.38
PbNAC94a	PbNAC9a	3	0.00278	1.64	2.35
PbNAC2e	PbNAC2f	4	0.00428	0.01	0.01
PbNAC83g	PbNAC83b	3	0.00494	2.91	4.75
PbNAC100d	PbNAC100a	3	0.00938	2.37	3.29
