# Supplementary figures and images for: Genome-wide analyses and expression patterns under abiotic stress of NAC transcription factors in white pear (Pyrus bretschneideri)
Source: BMC Plant Biol. 2019 Apr 25;19:161. doi: 10.1186/s12870-019-1760-8 (PMC6485137; doi:10.1186/s12870-019-1760-8)

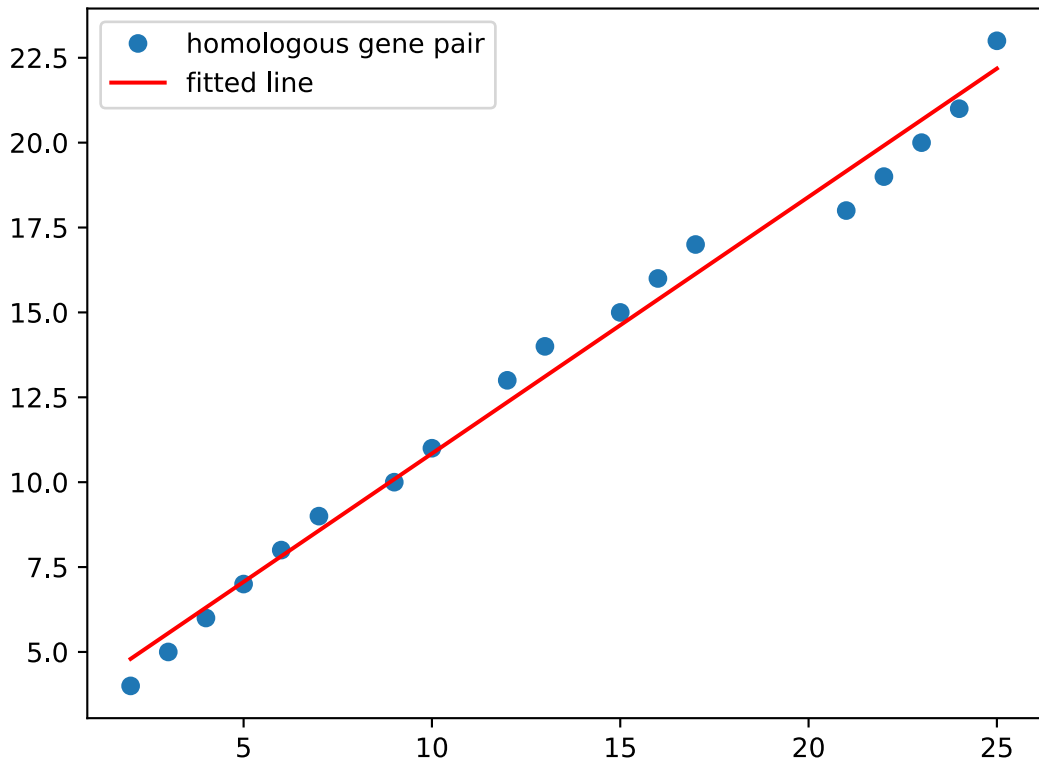

Supplement: Supplementary file 6 — Figure S1. Coefficients of determination of PbNAC73a and PbNAC73b .Figure S2. Segmental duplication between members of PbNAC73a and PbNAC73b. (ZIP 25 kb) [file 12870_2019_1760_MOESM6_ESM.zip › Additional file 6 Fig. S1.pdf]

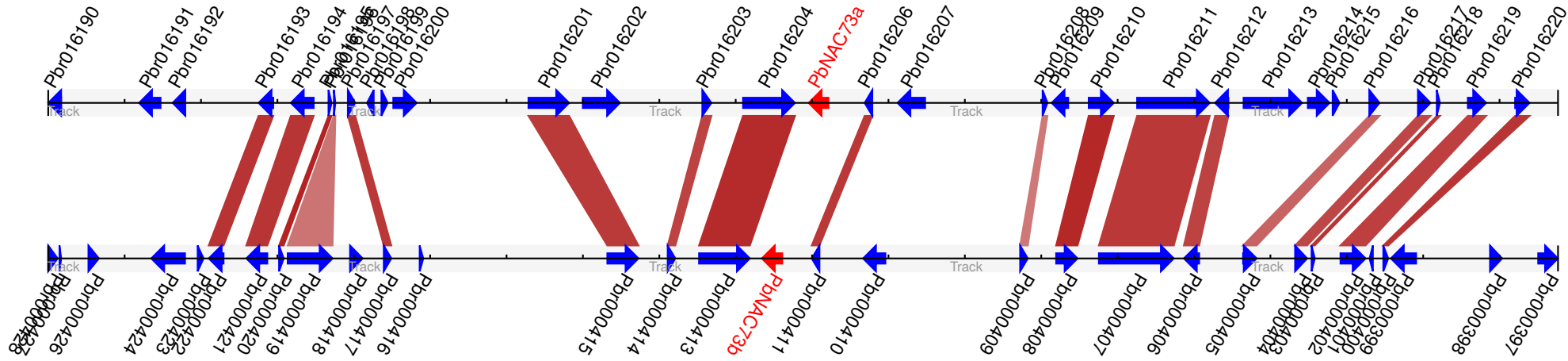

Supplement: Supplementary file 6 — Figure S1. Coefficients of determination of PbNAC73a and PbNAC73b .Figure S2. Segmental duplication between members of PbNAC73a and PbNAC73b. (ZIP 25 kb) [file 12870_2019_1760_MOESM6_ESM.zip › Additional file 6 Fig. S2.pdf]

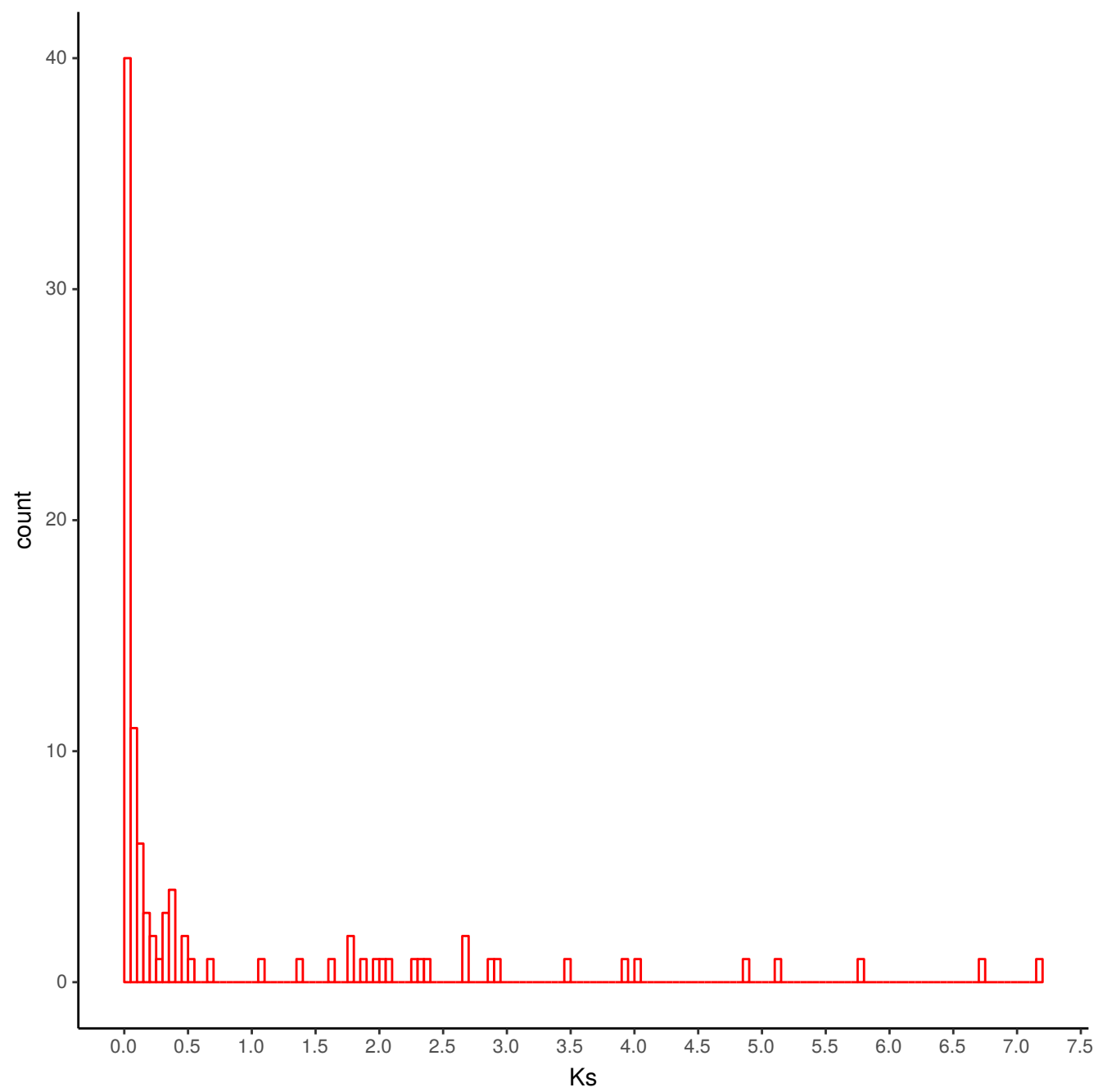

Supplement: Supplementary file 7 — Figure S3. Distribution of mean Ks values of PbNAC gene pairs in synteny blocks. (PDF 6 kb) [file 12870_2019_1760_MOESM7_ESM.pdf]
