# Supplementary material for: Genome-wide analyses and expression patterns under abiotic stress of NAC transcription factors in white pear (Pyrus bretschneideri)
Source: BMC Plant Biol. 2019 Apr 25;19:161. doi: 10.1186/s12870-019-1760-8 (PMC6485137; doi:10.1186/s12870-019-1760-8)
Supplement: Supplementary file 8 — Table S6 Ka/Ks related to genes in the NAC gene family in P. bretschneideri. (PDF 48 kb) [file 12870_2019_1760_MOESM8_ESM.pdf]

Duplicated gene 1	Duplicated gene 2	Ka	Ks	Ka/Ks
PbNAC83j	PbNAC83h	0.0071	0.0118	0.6017
PbNAC58a	PbNAC58c	0.04	0.1617	0.2474
PbNAC42a	PbNAC42d	0.0613	0.1198	0.5117
PbNAC7f	PbNAC7e	0	0.0054	-
PbNAC8a	PbNAC8b	0.0439	0.1706	0.2573
PbNAC73a	PbNAC73b	0.0217	0.1453	0.1493
PbNAC2j	PbNAC86b	0.0359	0.0887	0.4047
PbNAC7a	PbNAC7e	0.0192	0.1762	0.109
PbNAC37b	PbNAC37a	0	0.0094	-
PbNAC44b	PbNAC44a	0.0118	0.0203	0.5813
PbNAC7f	PbNAC7a	0.0192	0.1694	0.1133
PbNAC83g	PbNAC83h	0.0802	0.143	0.5608
PbNAC30a	PbNAC30b	0.0015	0	-
PbNAC7b	PbNAC7e	0.0177	0.1695	0.1044
PbNAC20b	PbNAC20c	0.0037	0.0127	0.2913
PbNAC7a	PbNAC7b	0.0013	0.0149	0.0872
PbNAC71b	PbNAC71a	0.007	0.0161	0.4348
PbNAC95	PbNAC57	0.0879	0.087	1.0103
PbNAC83g	PbNAC83j	0.0784	0.1283	0.6111
PbNAC7f	PbNAC7b	0.0177	0.1628	0.1087
PbNAC74a	PbNAC74b	0.0659	0.1698	0.3881
PbNAC100c	PbNAC100d	0.0796	0.2106	0.378
PbNAC94a	PbNAC94b	0.0429	0.2469	0.1738
PbNAC33b	PbNAC33a	0.0261	0.1292	0.202
PbNAC32d	PbNAC90f	0.0985	0.2399	0.4106
PbNAC90e	PbNAC90c	0.1802	0.4004	0.45
PbNAC34c	PbNAC34b	0.0383	0.0842	0.4549
PbNAC98b	PbNAC98a	0.0013	0	-
PbNAC20a	PbNAC20b	0.0389	0.1141	0.3409
PbNAC20a	PbNAC20c	0.0428	0.1292	0.3313
PbNAC98b	PbNAC98c	0.0487	0.3091	0.1576
PbNAC2j	PbNAC86a	0.0359	0.0887	0.4047
PbNAC98c	PbNAC98a	0.0501	0.3091	0.1621
PbNAC91b	PbNAC91a	0.0873	0.1683	0.5187
PbNAC103a	PbNAC82	0.0784	0.1754	0.447
PbNAC104a	PbNAC104c	0.024	0.1808	0.1327
PbNAC31b	PbNAC31a	0.0737	0.2352	0.3134
PbNAC28a	PbNAC28c	0.0389	0.1454	0.2675
PbNAC87b	PbNAC87a	0.0503	0.1099	0.4577
PbNAC87b	PbNAC47b	0.7798	2.4853	0.3138
PbNAC42f	PbNAC42e	0.0287	0.0814	0.3526
PbNAC25d	PbNAC25c	0.0684	0.16	0.4275
PbNAC38b	PbNAC38a	0.0346	0.1782	0.1942
PbNAC83a	PbNAC83b	0.0613	0.1765	0.3473
PbNAC40a	PbNAC40b	0.0154	0.0195	0.7897

PbNAC100b	PbNAC100a	0.0391	0.1855	0.2108
PbNAC32b	PbNAC32c	0.0715	0.1255	0.5697
PbNAC90d	PbNAC90c	0.1055	0.2158	0.4889
PbNAC28a	PbNAC28b	0.0389	0.1454	0.2675
PbNAC83i	PbNAC83h	0.0071	0.0118	0.6017
PbNAC104b	PbNAC104c	0	0.0073	-
PbNAC83j	PbNAC83i	0	0	-
PbNAC86a	PbNAC86b	0	0	-
PbNAC104a	PbNAC104b	0.024	0.172	0.1395
PbNAC83c	PbNAC83d	0.0057	0.0066	0.8636
PbNAC2g	PbNAC2h	0.0015	0.0101	0.1485
PbNAC94b	PbNAC9a	0.3255	1.5395	0.2114
PbNAC83c	PbNAC83e	0.0057	0.0066	0.8636
PbNAC25c	PbNAC25b	0.539	1.7055	0.316
PbNAC56a	PbNAC72a	0.6547	4.5562	0.1437
PbNAC56a	PbNAC56b	0.035	0.1919	0.1824
PbNAC29	PbNAC32d	0.9454	2.3572	0.4011
PbNAC25d	PbNAC25b	0.5387	1.4174	0.3801
PbNAC38d	PbNAC42b	1.1298	2.3217	0.4866
PbNAC51b	PbNAC78a	0.8284	1.7718	0.4675
PbNAC90a	PbNAC90c	0.4653	1.4769	0.3151
PbNAC83f	PbNAC83d	0.0642	0.2474	0.2595
PbNAC2h	PbNAC2m	1.2693	-1	-
PbNAC83f	PbNAC83e	0.0642	0.2474	0.2595
PbNAC30a	PbNAC30c	0.0015	0	-
PbNAC42c	PbNAC50	0.6528	1.8081	0.361
PbNAC83g	PbNAC83a	0.529	1.9221	0.2752
PbNAC90a	PbNAC90e	0.4595	2.2142	0.2075
PbNAC29	PbNAC90f	0.9312	-1	-
PbNAC83c	PbNAC83f	0.0642	0.2378	0.27
PbNAC56a	PbNAC72b	0.9475	2.4514	0.3865
PbNAC21a	PbNAC21b	0.003	0.0104	0.2885
PbNAC25a	PbNAC56b	0.3902	1.7447	0.2236
PbNAC25a	PbNAC72a	0.6639	-1	-
PbNAC42c	PbNAC42b	0.0707	0.2413	0.293
PbNAC21a	PbNAC2j	1.1458	-1	-
PbNAC83a	PbNAC83j	0.5293	3.2822	0.1613
PbNAC91b	PbNAC14a	0.7038	2.8086	0.2506
PbNAC2a	PbNAC2c	0.0906	0.4513	0.2008
PbNAC104b	PbNAC38e	1.0761	-1	-
PbNAC2g	PbNAC2i	0.0089	0.0306	0.2908
PbNAC34a	PbNAC34c	0.0517	0.1151	0.4492
PbNAC100c	PbNAC100a	0.2974	2.0876	0.1425
PbNAC43b	PbNAC43a	0.3472	3.1899	0.1088
PbNAC42c	PbNAC104b	0.6366	1.4362	0.4433
PbNAC83f	PbNAC83b	0.2462	1.5721	0.1566

PbNAC83a	PbNAC83h	0.5246	3.0289	0.1732
PbNAC21a	PbNAC103c	1.1701	2.4784	0.4721
PbNAC7d	PbNAC37a	0.5095	-1	-
PbNAC94a	PbNAC9a	0.3178	1.5066	0.2109
PbNAC2e	PbNAC2f	0	0	-
PbNAC83g	PbNAC83b	0.4583	1.9203	0.2387
PbNAC100d	PbNAC100a	0.304	1.8156	0.1674
