# Supplementary material for: Genome-wide analyses and expression patterns under abiotic stress of NAC transcription factors in white pear (Pyrus bretschneideri)
Source: BMC Plant Biol. 2019 Apr 25;19:161. doi: 10.1186/s12870-019-1760-8 (PMC6485137; doi:10.1186/s12870-019-1760-8)
Supplement: Supplementary file 9 — Table S7 Positive selection of current WGD/segmental duplication using branch-site model A. (PDF 68 kb) [file 12870_2019_1760_MOESM9_ESM.pdf]

Positive selection of current WGD/segmental duplication using branch-site model A	
**Gene name**	**Likelihood ratio test （LRT）p vlaue**
PbNAC58c	0.000000234
PbNAC90e	0.000000436
PbNAC83b	0.001820265
PbNAC91b	0.018308198
PbNAC100c	0.038211381
PbNAC83f	0.046947976
PbNAC83g	0.047039363
PbNAC74b	0.048348462
