# Supplementary material for: Genome-wide analyses and expression patterns under abiotic stress of NAC transcription factors in white pear (Pyrus bretschneideri)
Source: BMC Plant Biol. 2019 Apr 25;19:161. doi: 10.1186/s12870-019-1760-8 (PMC6485137; doi:10.1186/s12870-019-1760-8)
Supplement: Supplementary file 12 — Table S10 Primer details for genes selected for quantitative real-time PCR analysis from results of digital transcript abundance measurements. (PDF 50 kb) [file 12870_2019_1760_MOESM12_ESM.pdf]

Primer details for genes selected for quantitative real-time PCR analysis from results of digital transcript abundance measurements.

Gene ID	Forward primer (5'-3')	Reverse primer (5'-3')
PbNAC2a	GCTGGATGATTGGGTTCTGT	TGTTCTTGTTGCTGCTGAGG
PbNAC2b	TGGATGGCTTCTCCGATATT	ATGTCCTGCAACTGGTAGGG
PbNAC17	CGATTCAGGGATCTCTCTCG	CCTCATTGCCGTTTTGATTT
PbNAC21a	GCCTTGTTGGGATGAGAAAA	AGGACCTAGGGGACCTTCAA
PbNAC21b	GCCTTGTTGGGATGAGAAAA	AGGACCTAGGGGACCTTCAA
PbNAC25a	ACCAGCATACCCTTGAAACG	TAGTAGTAGCGGCAGCAGCA
PbNAC56b	AACAAGCCCAACAACAAACC	ACACAGCACCCAGTCATCAA
PbNAC72a	CCAAAGGCATCAAGACCAAT	ATTCATCCAACCTGGAGCTG
PbNAC72b	GGTACTGGAAGGCAACTGGA	TGAGGCGATACTCGTGCATA
PbNAC100b	ATTTCGATTCCATCCAACCA	CCTCCCCAATTGCTTTACAA
